# Therapist-Supported Internet-Delivered Exposure and Response Prevention for Children and Adolescents With Tourette Syndrome: A Randomized Clinical Trial

**DOI:** 10.1001/jamanetworkopen.2022.25614

**Published:** 2022-08-15

**Authors:** Per Andrén, Moa Holmsved, Helene Ringberg, Vera Wachtmeister, Kayoko Isomura, Kristina Aspvall, Fabian Lenhard, Charlotte L. Hall, E. Bethan Davies, Tara Murphy, Chris Hollis, Filipa Sampaio, Inna Feldman, Matteo Bottai, Eva Serlachius, Erik Andersson, Lorena Fernández de la Cruz, David Mataix-Cols

**Affiliations:** 1 Centre for Psychiatry Research, Department of Clinical Neuroscience, Karolinska Institutet, Stockholm, Sweden; 2 Stockholm Health Care Services, Region Stockholm, Stockholm, Sweden; 3 Mental Health and Clinical Neurosciences, School of Medicine, University of Nottingham, Queen’s Medical Centre, Nottingham, United Kingdom; 4 National Institute for Health and Care Research MindTech MedTech Co-operative, Institute of Mental Health, School of Medicine, Division of Psychiatry and Applied Psychology, University of Nottingham, Innovation Park, Triumph Road, Nottingham, United Kingdom; 5 National Institute for Health and Care Research Nottingham Biomedical Research Centre, Institute of Mental Health, Mental Health and Clinical Neurosciences, University of Nottingham, Innovation Park, Nottingham, United Kingdom; 6 Great Ormond Street Institute of Child Health, University College London, London, United Kingdom; 7 Psychological and Mental Health Services, Great Ormond Street Hospital for Children National Health Service Foundation Trust, Great Ormond Street, London, United Kingdom; 8 Department of Public Health and Caring Sciences, Uppsala University, Uppsala, Sweden; 9 Unit of Biostatistics, Institute of Environmental Medicine, Karolinska Institutet, Stockholm, Sweden; 10 Child and Adolescent Psychiatry, Department of Clinical Sciences, Medical Faculty, Lund University, Lund, Sweden

## Abstract

**Importance:**

The availability of behavior therapy for individuals with Tourette syndrome (TS) and chronic tic disorder (CTD) is limited.

**Objective:**

To determine the efficacy and cost-effectiveness of internet-delivered exposure and response prevention (ERP) for children and adolescents with TS or CTD.

**Design, Setting, and Participants:**

This single-masked, parallel group, superiority randomized clinical trial with nationwide recruitment was conducted at a research clinic in Stockholm, Sweden. Out of 615 individuals assessed for eligibility, 221 participants meeting diagnostic criteria for TS or CTD and aged 9 to 17 years were included in the study. Enrollment began in April 2019 and ended in April 2021. Data were analyzed between October 2021 and March 2022.

**Interventions:**

Participants were randomized to 10 weeks of therapist-supported internet-delivered ERP for tics (111 participants) or to therapist-supported internet-delivered education for tics (comparator group, 110 participants).

**Main Outcomes and Measures:**

The primary outcome was change in tic severity from baseline to the 3-month follow-up as measured by the Total Tic Severity Score of the Yale Global Tic Severity Scale (YGTSS-TTSS). YGTSS-TTSS assessors were masked to treatment allocation. Treatment response was operationalized as a score of 1 (“Very much improved”) or 2 (“Much improved”) on the Clinical Global Impression–Improvement scale.

**Results:**

Data loss was minimal, with 216 of 221 participants (97.7%) providing primary outcome data. Among randomized participants (152 [68.8%] boys; mean [SD] age, 12.1 [2.3] years), tic severity improved significantly, with a mean reduction of 6.08 points on the YGTSS-TTSS in the ERP group (mean [SD] at baseline, 22.25 [5.60]; at 3-month follow-up, 16.17 [6.82]) and 5.29 in the comparator (mean [SD] at baseline, 23.01 [5.92]; at 3-month follow-up, 17.72 [7.11]). Intention-to-treat analyses showed that the 2 groups improved similarly over time (interaction effect, −0.53; 95% CI, −1.28 to 0.22; *P* = .17). Significantly more participants were classified as treatment responders in the ERP group (51 of 108 [47.2%]) than in the comparator group (31 of 108 [28.7%]) at the 3-month follow-up (odds ratio, 2.22; 95% CI, 1.27 to 3.90). ERP resulted in more treatment responders at little additional cost compared with structured education. The incremental cost per quality-adjusted life-year gained was below the Swedish willingness-to-pay threshold, at which ERP had a 66% to 76% probability of being cost-effective.

**Conclusions and Relevance:**

Both interventions were associated with clinically meaningful improvements in tic severity, but ERP led to higher response rates at little additional cost.

**Trial Registration:**

ClinicalTrials.gov identifier: NCT03916055

## Key Points

**Question:**

Is therapist-supported, internet-delivered exposure and response prevention (ERP) efficacious and cost-effective for young people with Tourette syndrome or chronic tic disorder?

**Findings:**

In this randomized clinical trial of 221 youths with Tourette syndrome or chronic tic disorder who received either therapist-supported internet-delivered ERP or structured education, both groups significantly improved over time, with no between-group differences in tic severity. However, ERP was associated with significantly higher treatment response rates (47% vs 29%) at little additional cost.

**Meaning:**

Both internet-delivered ERP and structured education were associated with improvements in tic severity, but ERP led to higher treatment response rates.

## Teaser

This randomized clinical trial investigates the effectiveness of therapist-supported online exposure and response prevention treatment for children with Tourette syndrome and chronic tic disorder compared with therapist-supported online education courses for tics.

## Introduction

Clinical guidelines recommend behavior therapy (BT) as a first-line treatment for Tourette syndrome (TS) and chronic tic disorder (CTD),^1,2^ but its availability is very limited.^3,4^ Various formats of remote delivery have been proposed to improve access,^5,6,7^ including internet-delivered BT, where the treatment is delivered through self-help texts, illustrations, and videos with minimal therapist support.^8^ We developed an internet-delivered BT program for TS and CTD and evaluated its feasibility and preliminary efficacy in a pilot randomized clinical trial (RCT).^7^ Results from our trial showed that exposure and response prevention (ERP)^9^ was particularly well-suited to guided online delivery. Following these promising results, we designed 2 parallel RCTs in England and Sweden comparing therapist-supported internet-delivered ERP with a robust comparator: internet-delivered education. In the English RCT^10^—the Online Remote Behavioral Intervention for Tics (ORBIT) trial—ERP was superior to the comparator in reducing tic severity. This study presents the results of the Swedish RCT, including a health economic evaluation.

## Methods

### Trial Design

The study was a single-masked, parallel group, superiority RCT comparing therapist-supported internet-delivered ERP with therapist-supported internet-delivered education (comparator) for children and adolescents with TS or CTD. Participants were assessed at baseline, 3 and 5 weeks into treatment, at posttreatment, and at 3 months posttreatment (primary end point). Additional 6-month and 12-month follow-ups will be reported separately. The study setting was a research clinic within the Child and Adolescent Mental Health Services in Stockholm, Sweden. Ethical approval was obtained from the Swedish Ethical Review Authority. All participants and their legal guardians provided written informed consent to participate. The full study protocol is published^11^ and available in Supplement 1. The report follows the Consolidated Standards of Reporting Trials (CONSORT) and the Consolidated Health Economic Evaluation Reporting Standards (CHEERS) reporting guidelines.

### Participants

Participants were recruited across Sweden through clinician referrals and self-referrals. The study was advertised to health care services, patient organizations, and directly to the public. After an initial telephone screening, potentially eligible participants were invited to a full psychiatric assessment performed face-to-face or through videoconference by trained researchers. Tic severity and impairment were assessed with the Yale Global Tic Severity Scale (YGTSS),^12^ and psychiatric comorbidities with the Mini-International Neuropsychiatric Interview for children and adolescents (MINI-KID).^13^ Eligible participants were between ages 9 and 17 years with a *Diagnostic and Statistical Manual of Mental Disorders (Fifth Edition)* (*DSM-5*) diagnosis of TS or CTD (eMethods 1 in Supplement 2).^14^

### Randomization and Allocation Concealment

Participants were randomized 1:1 to ERP or the comparator through an online service (Randomize.net) that was set up and monitored by an independent clinical trials unit (Karolinska Trial Alliance). The randomization sequence, using randomly varying block sizes, was concealed from the study team. Several researchers performed the randomization, enrolled participants, and assigned participants to treatments and therapists according to a task delegation list. Participants were informed that they would receive 1 of 2 behavioral interventions for TS and CTD but were not given details about any of the interventions’ content. The principal investigator, outcome assessors, statistician, and health economists were masked to group allocation throughout the trial (eMethods 2 in Supplement 2).

### Interventions

Both interventions were delivered through an internet platform over 10 weeks. Children and parents each had separate logins to 10 chapters including overlapping treatment content in the form of self-help texts, illustrations, videos, worksheets, exercises, and homework assignments. Treatment completion was defined a priori as the completion of the first 4 child chapters, which contained the core ingredients of each intervention. Screenshots of the interventions are displayed in eFigure 1 in Supplement 2.

In both interventions, children and parents were supported by a designated therapist via asynchronous text messages inside the platform, supplemented by telephone calls when needed, through all 10 weeks. The role of the therapist was to provide feedback, answer questions, and encourage treatment adherence. Children and parents could write to their therapist at any time, while the therapist provided support at least every 48 hours (on workdays). The therapists were clinical or trainee psychologists with specific BT training (Supplement 1).

The content of the active ERP intervention was based on existing manuals.^9,15^ The primary focus of the intervention was to practice tic suppression (response prevention) and gradually provoke premonitory urges (ie, unpleasant sensations typically preceding tics) to make the tic suppression more challenging (exposure). The active comparator was based on control interventions used in previous RCTs of BT for TS and CTD.^16,17^ It consisted of education about TS and CTD and common comorbid disorders, as well as behavioral exercises (eg, engaging in healthy habits, sharing information about TS and CTD with peers), and was designed to match the ERP intervention in every respect, except for its core components. Further details on both interventions are presented elsewhere^11^ and in Supplement 1.

### Outcome Measures

The primary outcome was tic severity as measured by the Total Tic Severity Score of the Yale Global Tic Severity Scale (YGTSS-TTSS), a clinician-rated semi-structured interview assessed on a 50-point scale (eMethods 3 in Supplement 2).^12^ Clinician-rated secondary outcome measures were the YGTSS Impairment score,^12^ the Children’s Global Assessment Scale (CGAS),^18^ the internet intervention Patient Adherence Scale (iiPAS),^19^ and the Clinical Global Impression Severity and Improvement (CGI-S/I) scales.^20^ Following previous studies,^16,17^ treatment response was operationalized as a CGI-I score of 1 (“Very much improved”) or 2 (“Much improved”). All clinician-rated measures were administered by masked assessors face-to-face at the clinic (posttreatment, 13%; 3-month follow-up, 6%), via videoconference (86%; 89%), or via telephone (1%; 5%). Follow-up assessments were primarily administered to both the child and at least 1 parent, but in a few cases only the child (in less than 1% of cases both posttreatment and at 3-month follow-up) or only a parent (3%; 8%) participated.

Self-reported and parent-reported outcome measures were completed online and included the Parent Tic Questionnaire (PTQ),^21^ the Child and Adolescent Gilles de la Tourette Syndrome–Quality of Life (C&A-GTS-QOL) scale,^22^ the Obsessive-Compulsive Inventory–Child Version (OCI-CV),^23^ the Short Mood and Feelings Questionnaire child- and parent-reported versions (SMFQ-C and SMFQ-P, respectively),^24^ the Side Effects Questionnaire,^25^ and the Working Alliance Inventory child- and parent-reported versions (WAI-C and WAI-P, respectively).^26^ Participants also answered questionnaires developed by the study team assessing treatment credibility, treatment satisfaction, and perceived need for further TS or CTD treatment. Other process outcomes included number of completed chapters, proportion of treatment completers, therapist support time, and masked assessors’ guesses of group allocation. Outcome measures and times of administration are available in Supplement 1.

### Safety Procedures

Adverse events were monitored with the parent-reported Side Effects Questionnaire,^25^ the SMFQ-C, a self-reported suicidality item, and regular contact between participants and trial staff. Safety aspects and data quality were externally monitored by the Karolinska Trial Alliance.

### Power Analysis

We estimated the power for the change in median YGTSS-TTSS between the 2 treatment groups from baseline to the primary end point. Two hundred participants were required to detect a statistically significant change in medians of 3 points on the YGTSS-TTSS with a power of 97%. We increased the sample size to 220 patients to account for a potential 10% dropout rate (eMethods 4 in Supplement 2).

### Statistical Analyses

The full statistical analysis plan (SAP) was decided a priori and can be found in the study protocol^11^ and Supplement 1. Due to the ordinal nature of the primary outcome measure, median differences were estimated. Intention-to-treat, linear quantile mixed models provided estimates and confidence intervals for the median differences.^27,28,29^ The model included the intercept, the binary treatment variable (ie, ERP and the comparator), the numeric time variable (baseline, posttreatment, 3-month follow-up), and the treatment-by-time interaction term. To enable comparisons with previous trials estimating mean differences, a complementary analysis using a linear mixed model was also performed according to our a priori SAP. Secondary outcomes were analyzed with linear quantile mixed models, complementary linear mixed models, quantile regression, logistic regression, and χ^2^ tests. All secondary analyses, unless otherwise specified, were intention-to-treat. The magnitude of the effects is presented as between-group differences in median relative to the interquartile range (for median differences) and as standardized between-group effect sizes (for mean differences, Cohen *d*).^30^ An α level of .05 was used throughout the study. Analyses were performed using Stata version 14.2 (StataCorp) and R version 4.1.1 (R Project for Statistical Computing).

The health economic evaluation was performed using 3 perspectives: (1) a health care organization perspective (including direct treatment costs for the clinic; ie, therapist time), (2) a health care sector perspective (additionally including health care resource use outside the clinic and medication costs), and (3) a societal perspective (additionally including costs beyond health care; eg, parents’ absenteeism from work). Resources used were collected with the parent-reported Trimbos/iMTA (Institute for Medical Technology Assessment) questionnaire for costs associated with psychiatric illness (TiC-P).^31^ Quality of life was estimated with the KIDSCREEN-10,^32^ which was mapped to the Child Health Utility 9D (CHU9D) to obtain quality-adjusted life-years (QALYs).^33^ For each of the 3 perspectives, a cost-effectiveness analysis (using treatment response rate as the outcome) and a cost-utility analysis (using QALYs as the outcome) were performed.^34^ Incremental cost-effectiveness ratios (ICERs) expressed as the cost per additional treatment responder or QALY were estimated. Further details are available in Supplement 1 and eMethods 5 in Supplement 2.

## Results

### Participants

**Figure 1.**
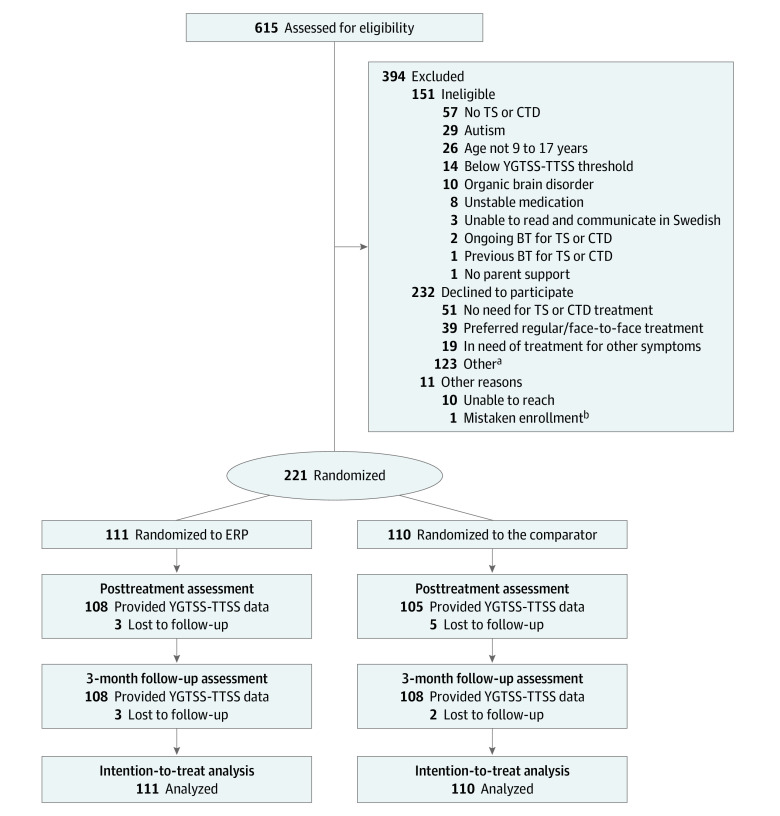
Flow Diagram of Study Participation BT indicates behavior therapy; CTD, chronic tic disorder; ERP, therapist-supported internet-delivered exposure with response prevention for children and adolescents with Tourette syndrome or chronic tic disorder; TS, Tourette syndrome; YGTSS-TTSS, Yale Global Tic Severity Scale–Total Tic Severity Score. ^a^Other included families that did not want to participate in a research study, did not feel motivated, did not have the energy for assessment or treatment, did not have enough time, did not want to be video recorded, did not want a TS or CTD diagnosis to be recorded in their patient record, with parents who wanted to participate but not the child, as well as cases where no specific reasons were specified.

Participant enrollment began on April 26, 2019, and ended on April 9, 2021. The final participant reached the 3-month follow-up (primary end point) on September 24, 2021. Out of 615 individuals assessed for eligibility, 221 were included in the study and randomized to the ERP group (111 participants) or the comparator (110 participants) (Figure 1). Participants had a mean (SD) age of 12.1 (2.3) years, and 152 participants (68.8%) were boys (Table 1). The most common tic disorder was TS (202 participants [91.4%]). Eighty-four participants (38.0%) had at least 1 comorbid diagnosis, the most common being attention-deficit/hyperactivity disorder (34 participants [15.4%]) and anxiety disorders (31 participants [14.0%]). A majority (189 participants [85.5%]) were unmedicated at baseline. Additional participant characteristics are presented in eTable 1 in Supplement 2. One treatment-unrelated serious adverse event (meningitis requiring hospitalization) was recorded in the comparator group (eResults 4 and eTable 12 in Supplement 2).

**Table 1.** Baseline Demographics and Clinical Characteristics for Study Participants

Characteristic	Participants, No. (%)		
	ERP (n = 111)	Comparator (n = 110)	Total (N = 221)
Age, mean (SD) [range], y	12.0 (2.3) [9-17]	12.1 (2.3) [9-17]	12.1 (2.3) [9-17]
Gender			
Boys	71 (64.0)	81 (73.6)	152 (68.8)
Girls	39 (35.1)	29 (26.4)	68 (30.8)
Other	1 (0.9)	0	1 (0.5)
Age of tic onset, mean (SD) [range], y	5.7 (2.0) [2-11]	6.2 (2.1) [2-14]	5.9 (2.1) [2-14]
Tic disorder			
Tourette syndrome	104 (93.7)	98 (89.1)	202 (91.4)
Chronic tic disorder			
Motor	7 (6.3)	9 (8.2)	16 (7.2)
Vocal	0	3 (2.7)	3 (1.4)
Comorbidity			
Any	44 (39.6)	40 (3.6)	84 (38.0)
Attention-deficit/hyperactivity disorder	20 (18.0)	14 (12.7)	34 (15.4)
Anxiety disorder	16 (14.4)	15 (13.6)	31 (14.0)
Obsessive-compulsive disorder	11 (9.9)	6 (5.5)	17 (7.7)
Depression	1 (0.9)	3 (2.7)	4 (1.8)
Other^a^	7 (6.3)	3 (2.7)	10 (4.5)
Medication status			
None	94 (84.7)	95 (86.4)	189 (85.5)
Melatonin	8 (7.2)	9 (8.6)	17 (7.7)
ADHD medication^b^	9 (8.1)	5 (4.5)	14 (6.3)
α-2 agonist	4 (3.6)	1 (0.9)	5 (2.3)
SSRI	2 (1.8)	3 (2.7)	5 (2.3)
Antipsychotic	1 (0.9)	1 (0.9)	2 (0.9)
Antihistaminic	0	2 (1.8)	2 (0.9)
Distance to the clinic, mean (SD), [range], km	220 (251) [ 0-1226]	194 (219) [1-869]	207 (235) [0-1226]
Highest level of parental education^c^			
Primary school	0	3 (2.7)	3 (1.4)
Secondary school	17 (15.3)	19 (17.3)	36 (16.3)
College/university			
<2 y	12 (10.8)	6 (5.5)	18 (8.1)
≥2 y	78 (70.2)	80 (72.7)	158 (71.5)
Postgraduate education	4 (3.6)	2 (1.8)	6 (2.7)
Parental occupation^c^			
Working	101 (91.0)	104 (94.5)	205 (92.8)
Student	4 (3.6)	3 (2.7)	7 (3.2)
Other^d^	6 (5.4)	3 (2.7)	9 (4.1)
Previous contact with health care services for TS or CTD	76 (68.5)	64 (58.2)	140 (63.3)
Previous BT for TS or CTD	12 (10.8)	9 (8.2)	21 (9.5)

Abbreviations: ADHD, attention-deficit/hyperactivity disorder; BT, behavior therapy; CTD, chronic tic disorder; ERP, therapist-supported internet-delivered exposure with response prevention for children and adolescents with Tourette syndrome or chronic tic disorder; SSRI, selective serotonin reuptake inhibitor; TS, Tourette syndrome.

^a^ Includes dyscalculia, dyslexia, excoriation (skin-picking) disorder, gender dysphoria, hoarding disorder, language disorder, and oppositional defiant disorder.

^b^ Includes stimulants and atomoxetine.

^c^ Primary parent supporting the treatment.

^d^ Unemployed, sick leave, or retired.

### Primary Outcome

Data loss on the YGTSS-TTSS was minimal, with 8 (3.6%) missing data points at posttreatment and 5 (2.3%) missing data points at the 3-month follow-up (Table 2 and Figure 1). The mean YGTSS-TTSS reduction from baseline to the 3-month follow-up was 6.08 raw points for the ERP group and 5.29 for the comparator, which were significant reductions in within-group linear quantile mixed model analyses (ERP: coefficient, −3.00; 95% CI, −3.63 to −2.37; *P* < .001; comparator: coefficient, −2.20; 95% CI, −2.90 to −1.50; *P* < .001) (eTable 2 in Supplement 2). Bootstrapped within-group effect sizes (medians relative the interquartile range) were 0.60 (95% CI, 0.42 to 0.78) for ERP and 0.44 (95% CI, 0.28 to 0.59) for the comparator. The between-group linear quantile mixed model found no significant interaction effect between group (ERP and the comparator) and time (baseline to the 3-month follow-up) on the YGTSS-TTSS (coefficient, −0.53; 95% CI, −1.28 to 0.22; *P* = .17) (Table 2). A planned complementary linear mixed model showed a similar result (coefficient, −0.39; 95% CI, −1.08 to 0.31; *P* = .28) (eTable 3 in Supplement 2).

**Table 2.** Results of Linear Quantile Mixed Models for the Primary Outcome and a Selection of Secondary Outcomes

Outcome	Participants with available data, No.	ERP (n = 111)^a^		Comparator (n = 110)^a^		Intention-to-treat linear quantile mixed model		
		Median (IQR)	Mean (SD)	Median (IQR)	Mean (SD)	Coefficient (95% CI)^b^	*P* value	Effect size (95% CI)^b^^,^^c^
**YGTSS-TTSS**								
Baseline	221	23 (18 to 26)	22.25 (5.60)	24 (19 to 27)	23.01 (5.92)	NA	NA	NA
Posttreatment	213	19 (13 to 23)	18.53 (5.94)	20 (15 to 24)	19.27 (7.20)	NA	NA	NA
3-mo follow-up^d^	216	17 (11 to 21)	16.17 (6.82)	19 (12 to 23)	17.72 (7.11)	−0.53 (−1.28 to 0.22)	.17	0.11 (−0.09 to 0.30)
**YGTSS Impairment**								
Baseline	221	20 (10 to 20)	18.38 (7.08)	20 (10 to 20)	18.73 (7.79)	NA	NA	NA
Posttreatment	213	10 (0 to 20)	10.65 (8.68)	10 (0 to 20)	11.52 (9.59)	NA	NA	NA
3-mo follow-up^d^	216	10 (0 to 10)	7.68 (8.82)	10 (0 to 10)	8.70 (8.10)	−0.26 (−1.70 to 1.18)	.72	0.05 (−0.34 to 0.44)
**CGI-S**								
Baseline	221	4 (4 to 5)	4.08 (0.74)	4 (4 to 5)	4.19 (0.72)	NA	NA	NA
Posttreatment	213	4 (3 to 4)	3.50 (0.86)	4 (3 to 4)	3.69 (0.91)	NA	NA	NA
3-mo follow-up^d^	216	3 (3 to 4)	3.24 (0.92)	4 (3 to 4)	3.49 (0.90)	−0.36 (−0.67 to −0.04)	.03^e^	0.71 (0.05 to 1.37)
**PTQ**								
Baseline	221	32 (19 to 44)	34.33 (19.06)	34 (21 to 51)	38.04 (23.27)	NA	NA	NA
Midtreatment^f^	210	22 (13 to 39)	25.73 (16.14)	26 (15 to 41)	29.83 (18.82)	NA	NA	NA
Posttreatment	214	17 (10 to 30)	21.08 (15.75)	19.5 (11 to 36.5)	25.05 (18.18)	NA	NA	NA
3-mo follow-up^d^	211	14 (6 to 25)	19.84 (17.92)	19 (7.5 to 37.5)	23.51 (18.14)	0.13 (−1.43 to 1.68)	.87	−0.01 (−0.22 to 0.19)
**C&A-GTS-QOL**								
Baseline	221	27 (17 to 39)	29.11 (15.06)	27.5 (18 to 43)	30.54 (16.54)	NA	NA	NA
Posttreatment	212	15 (8 to 28.5)	19.68 (15.48)	20.5 (12 to 31)	22.86 (15.71)	NA	NA	NA
3-mo follow-up^d^	208	16 (8 to 28)	19.76 (16.26)	17 (9 to 27)	20.05 (15.72)	0.46 (−1.63 to 2.55)	.67	−0.04 (−0.24 to 0.16)

Abbreviations: C&A-GTS-QOL, Child and Adolescent Gilles de la Tourette Syndrome–Quality of life scale; CGI-S, Clinical Global Impression–Severity scale; ERP, therapist-supported internet-delivered exposure with response prevention for children and adolescents with Tourette syndrome or chronic tic disorder; PTQ, Parent Tic Questionnaire; YGTSS, Yale Global Tic Severity Scale; YGTSS-TTSS, Yale Global Tic Severity Scale–Total Tic Severity Score.

^a^ Observed values calculated from completer data.

^b^ Estimates (negative or positive) compare with the comparator as the reference point.

^c^ Bootstrapped effect sizes, interpreted as between-group differences in median relative the interquartile range, are derived from the linear quantile mixed models.

^d^ Primary end point.

^e^ Significant at an α = .05.

^f^ Five weeks into treatment.

### Secondary Outcomes

At the primary end point (3-month follow-up), significantly more participants were classified as treatment responders in the ERP group (51 participants [47.2%]) than in the comparator (31 participants [28.7%]) (odds ratio, 2.22; 95% CI, 1.27 to 3.90; *P* = .005) (eTable 4 in Supplement 2). Within-group linear quantile mixed model analyses showed that both groups improved from baseline to the 3-month follow-up on the YGTSS Impairment score, the PTQ, the C&A-GTS-QOL, the CGAS, the OCI-CV, the SMFQ-C, and the SMFQ-P (eTable 2 in Supplement 2). Furthermore, there were improvements on the CGI-S and the parent-reported KIDSCREEN-10 in the ERP group only. A between-group linear quantile mixed model analysis identified interaction effects between group (ERP and the comparator) and time (baseline to the 3-month follow-up) on the CGI-S and the parent-reported KIDSCREEN-10, both in favor of ERP (Table 2; eTable 5 in Supplement 2). The results of the complementary linear mixed model analyses are shown in eTable 3 in Supplement 2.

### Process Outcomes

Out of the 10 treatment chapters, children completed a mean (SD) of 8.9 (1.8) chapters in the ERP group and 8.7 (2.2) in the comparator group. For parents, mean chapters completed were 8.9 (1.7) for ERP and 8.8 (2.3) for the comparator. All 111 participants in the ERP group and 104 (94.5%) of the participants in the comparator group were classified as treatment completers. Mean (SD) therapist support time (text messages [96%] and telephone [4%]) was 19.1 (5.8) minutes per participant and week in the ERP group and 16.6 (6.5) minutes per participant and week in the comparator group, a statistically significant difference (*t* = −3.01; *P* = .003). Three weeks into treatment, both children and parents rated the ERP intervention to be more credible than the comparator (coefficient, 1; 95% CI, 0.38 to 1.62; *P* = .002). At the same assessment point, children also rated the patient-therapist working alliance, as measured by the WAI-C, to be higher in ERP than in the comparator (coefficient, 3; 95% CI, 0.95 to 5.05; *P* = .004), while no between-group difference was identified for the equivalent parent rating (WAI-P). Both children and parents were more satisfied with ERP than with the comparator (children: coefficient, 3; 95% CI, 1.13 to 4.87; *P* = .002; parents: coefficient, 4; 95% CI, 2.33 to 5.66; *P* < .001). Further details on process outcomes are presented in eResults 1 and eTable 6 in Supplement 2. Masked assessors were better than chance at guessing the correct group allocation (61%; χ^2^ = 10.49; *P* = .001), but guesses were not significantly associated with treatment outcomes (eResults 2 and eTable 7 in Supplement 2).

### Post hoc Analyses

Seven participants (3.2%) deviated from protocol (ie, received another behavioral intervention or changed medication) and were excluded in a sensitivity analysis, which showed similar results to those in the main analysis (eResults 3 in Supplement 2). Additional post hoc analyses found that ERP was superior to the comparator for older and male participants, but not for younger and female participants (eTables 8-11 in Supplement 2).

### Health Economic Evaluation

One participant in the comparator was excluded from the economic evaluation because of a serious adverse event, which could have skewed the cost estimates. Baseline KIDSCREEN-10 (child version) scores, CHU9D utility scores, total health care costs, total societal costs, and unit costs are presented in eTables 13 and 14 in Supplement 2.

The mean (SE) intervention costs (ie, the therapist-support time, referred to as the health care organization perspective) were slightly higher for the ERP group ($117.38 [8.78]) than the comparator ($102.23 [3.65]; mean difference, $15.14; 95% CI, $5.08 to $25.20) (eTable 15 in Supplement 2). From baseline to the 3-month follow-up, the ERP group showed higher costs for the health care sector perspective, although this difference was not significant (adjusted mean difference; $91.30; 95% CI, −$64.48 to $452.31) and the societal perspective (adjusted mean difference, $26.56; 95% CI, −$404.46 to $976.75) (eTable 15 in Supplement 2).

**Figure 2.**
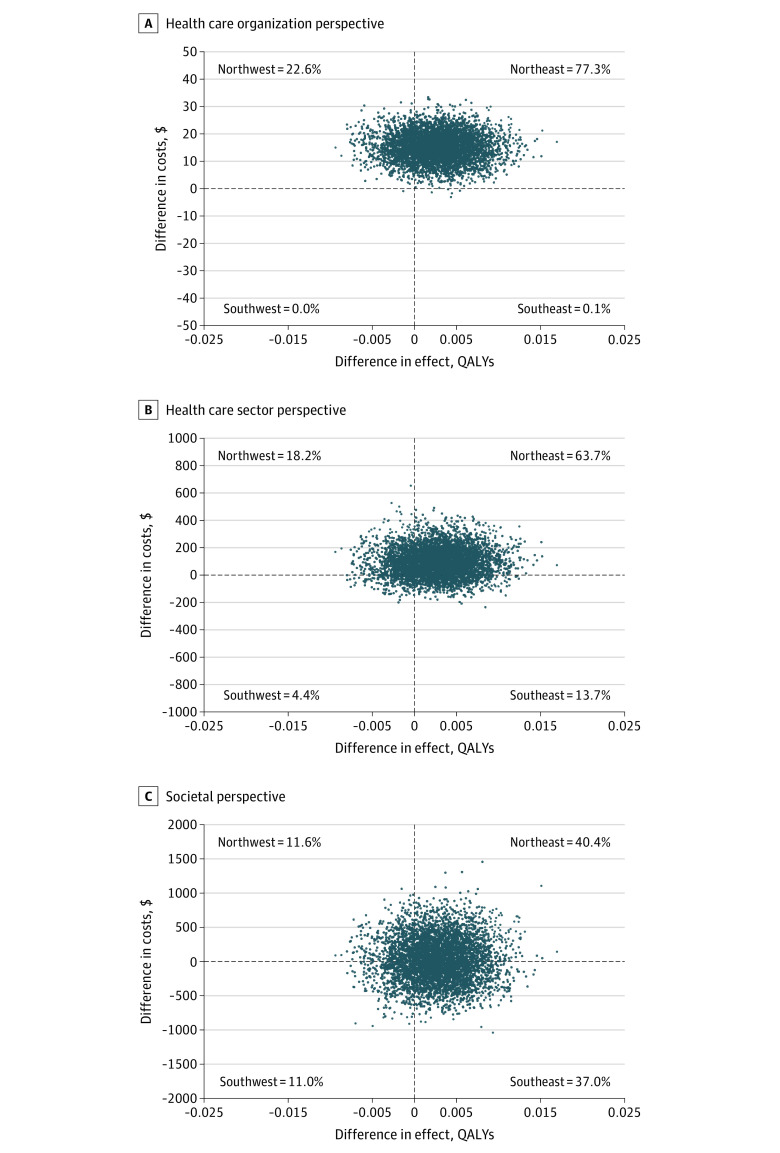
Cost-effectiveness Planes With QALYs as the Outcome for 3 Costing Perspectives All 3 cost-effectiveness planes compare ERP with therapist-supported internet-delivered education for children and adolescents with Tourette syndrome or chronic tic disorder (ie, the comparator) using QALYs as the outcome. In panel A, the health care organization perspective includes costs of the ERP or comparator interventions (ie, the therapist-support time). In panel B, the health care sector perspective includes costs of the ERP or comparator interventions, health care visits, and medication or supplements. In panel C, the societal perspective includes costs of the ERP or comparator interventions, health care visits, medication or supplements, and other sector costs (eg, productivity losses, child school absenteeism). ERP indicates therapist-supported internet-delivered exposure with response prevention for children and adolescents with Tourette syndrome or chronic tic disorder; QALY, quality-adjusted life-year.

The cost-effectiveness analysis showed statistically significant higher treatment response rates for the ERP group than the comparator at slightly higher costs (eTable 17 and eFigure 2 in Supplement 2). In the cost utility analysis, mean CHU9D utility scores per assessment point and total QALYs for the study period are presented in eTable 16 in Supplement 2. This analysis showed small nonsignificant gains in QALYs for the ERP group at higher costs (eTable 17 in Supplement 2; Figure 2). The ICERs for the different perspectives varied between $79 and $476 per additional treatment responder and between $5496 and $33 138 per QALY gained. The ICER estimates were under the threshold of $79 000, a reported willingness-to-pay per 1 QALY in Swedish society.^35^ Depending on the perspective, the probability of ERP being cost-effective ranged from 66% to 76% at that threshold (eFigure 3 and eFigure 4 in Supplement 2).

## Discussion

In this RCT, 221 children and adolescents with TS or CTD received 10 weeks of either therapist-supported internet-delivered ERP or therapist-supported internet-delivered education. Participants in both groups improved meaningfully and similarly across a range of measures, with no significant interaction effect on tic severity from baseline to the primary end point. However, treatment response rates (47% vs 29%) and satisfaction were significantly higher in the ERP group. Strengths of the study included the use of an active comparator, nationwide recruitment, a large sample size, and very low data attrition. Furthermore, the study had thorough therapist and assessor training procedures, transparent masking and safety procedures, and external monitoring.

The ERP group improved similarly to the identical ERP group in the parallel ORBIT trial,^10^ but slightly less than the Comprehensive Behavioral Intervention for Tics (CBIT) group in the largest RCT of face-to-face BT (decrease of 6.1 vs 7.6 raw YGTSS-TTSS points at their primary end points, respectively).^16^ We found larger-than-expected improvements in tic severity in the comparator group (decrease of 5.3 raw YGTSS-TTSS points at the primary end point), which were not observed in previous trials.^10,16^ It is unlikely that these improvements were solely due to spontaneous fluctuations in tic severity, because such large improvements have not been observed in pure wait-listed conditions in previous trials of BT for TS and CTD,^5,36^ including a recent pediatric RCT of internet-delivered CBIT.^37^ Participant selection may have played a role in these findings; participants in the current study were highly educated, less severe, less frequently on TS or CTD medication, and had lower rates of comorbid psychiatric disorders than those in other large clinical trials of BT for TS or CTD.^10,16^ Furthermore, the current study employed more experienced therapists than those in ORBIT,^10^ which may have made the comparator more potent than that of the ORBIT trial.

Post hoc analyses revealed significant group by time interaction effects on the YGTSS-TTSS for older participants (ages 12 to 17 years), but not for younger participants (ages 9 to 11 years). This result suggests younger participants benefitted more from structured education alone. This aligns well with the traditional view of providing psychoeducation to families of young children with tics while adopting a “wait and see” approach.^2^ We also found that while ERP was superior to education for boys, no such effect was evident among girls. These post hoc results should be interpreted cautiously due to the relatively small number of girls in the trial, but suggest that further research on potential sex differences in response to digital interventions for tics is needed.

Our economic analyses showed higher intervention costs for the ERP group, but no significant differences in other health care or societal costs. Cost-effectiveness analysis showed that ERP resulted in significantly more treatment responders at little additional cost. In the cost-utility analysis, no significant difference in QALYs was found. The ICERs were below the cost-effectiveness threshold of $79 000 per QALY,^35^ at which ERP had a 66% to 76% probability of being cost-effective, depending on the costing perspective.

As a whole, our findings suggest that both internet-delivered interventions could be implemented into regular health care to increase treatment access for children and adolescents with TS or CTD. However, we would favor the implementation of ERP based on its higher treatment response rates, likely cost-effectiveness, superior working alliance and satisfaction ratings, as well as the results from the parallel ORBIT trial.^10^

### Limitations

This study had several limitations. First, there was an absence of a third wait-listed group to control for the natural passage of time. Second, the inclusion of a generally mild group of participants may have somewhat diluted between group differences. Third, the exclusion of participants with comorbid autism may have limited the generalizability of the findings. Fourth, the short time frame of the health economic evaluation may not fully capture the societal costs associated with the disorder.

## Conclusions

Therapist-supported internet-delivered ERP and education were both associated with significantly and clinically meaningful improvements in tic severity, although treatment response rates and satisfaction were significantly higher in the ERP group. Implementation of the digital ERP intervention into regular health care would increase availability of treatment for young people with TS or CTD.

## Notes

Supplement 1.
Trial Protocol and Statistical Analysis Plan

Supplement 2. **eMethods 1.** Eligibility Criteria **eMethods 2.** Masking Procedures **eMethods 3.** Primary Outcome, Including Inter-Rater Reliability **eMethods 4.** Power Analysis **eMethods 5.** Further Details on the Health Economic Evaluation **eResults 1.** Process Outcomes **eResults 2.** Masking Integrity **eResults 3.** Post hoc Analyses **eResults 4.** Adverse Events **eTable 1.** Additional Baseline Demographics and Clinical Characteristics for Study Participants **eTable 2.** Results of Within-Group Linear Quantile Mixed Models for the Primary and Secondary Outcomes, From Baseline to the 3-Month Follow-up **eTable 3.** Results of Complementary Between-Group Linear Mixed Models for the Primary and Secondary Outcomes, From Baseline to the 3-Month Follow-up **eTable 4.** Treatment Response Rates at Posttreatment (Post hoc) and the 3-Month Follow-up **eTable 5.** Results of Between-Group Linear Quantile Mixed Models for Additional Secondary Outcomes **eTable 6.** Results of *t* Tests and Quantile Regressions for Process Outcomes **eTable 7.** Group Allocation Guesses and Motivations, by Assessors **eTable 8.** Post hoc Analyses Split by Median Age, Age Group 9-11 Years **eTable 9.** Post hoc Analyses Split by Median Age, Age Group 12-17 Years **eTable 10.** Post hoc Analyses Split by Gender, Boys **eTable 11.** Post hoc Analyses Split by Gender, Girls **eTable 12.** Reported Adverse Events Between Baseline and the 3-Month Follow-up **eTable 13.** Health Economic Evaluation, Baseline Outcomes and Costs (2021 USD) **eTable 14.** Unit Costs Used in the Costing of Resources, in 2021 USD **eTable 15.** Cost Means and Differences From Baseline to the 3-Month Follow-up, After Multiple Imputation, in 2021 USD **eTable 16.** Mean CHU9D Utility Scores per Assessment Point and Total QALYs Over the Study Period, After Multiple Imputation **eTable 17.** Outcomes and Costs From Baseline to the 3-Month Follow-up, After Multiple Imputation **eFigure 1.** Screenshots of the Internet Platform and Interventions **eFigure 2.** Cost-effectiveness Planes With Treatment Response as the Outcome for Three Costing Perspectives **eFigure 3.** Cost-effectiveness Acceptability Curves With Treatment Response as the Outcome for Three Costing Perspectives **eFigure 4.** Cost-effectiveness Acceptability Curves With QALYs as the Outcome for Three Costing Perspectives

Supplement 3.
Data Sharing Statement
