# Supplementary material for: Therapist-Supported Internet-Delivered Exposure and Response Prevention for Children and Adolescents With Tourette Syndrome: A Randomized Clinical Trial
Source: JAMA Netw Open. 2022 Aug 15;5(8):e2225614. doi: 10.1001/jamanetworkopen.2022.25614 (PMC9379743; doi:10.1001/jamanetworkopen.2022.25614)
Supplement: Supplement 2. — eMethods 1. Eligibility Criteria eMethods 2. Masking Procedures eMethods 3. Primary Outcome, Including Inter-Rater Reliability eMethods 4. Power Analysis eMethods 5. Further Details on the Health Economic Evaluation eResults 1. Process Outcomes eResults 2. Masking Integrity eResults 3. Post hoc Analyses eResults 4. Adverse Events eTable 1. Additional Baseline Demographics and Clinical Characteristics for Study Participants eTable 2. Results of Within-Group Linear Quantile Mixed Models for the Primary and Secondary Outcomes, From Baseline to the 3-Month Follow-up eTable 3. Results of Complementary Between-Group Linear Mixed Models for the Primary and Secondary Outcomes, From Baseline to the 3-Month Follow-up eTable 4. Treatment Response Rates at Posttreatment (Post hoc) and the 3-Month Follow-up eTable 5. Results of Between-Group Linear Quantile Mixed Models for Additional Secondary Outcomes eTable 6. Results of t Tests and Quantile Regressions for Process Outcomes eTable 7. Group Allocation Guesses and Motivations, by Assessors eTable 8. Post hoc Analyses Split by Median Age, Age Group 9-11 Years eTable 9. Post hoc Analyses Split by Median Age, Age Group 12-17 Years eTable 10. Post hoc Analyses Split by Gender, Boys eTable 11. Post hoc Analyses Split by Gender, Girls eTable 12. Reported Adverse Events Between Baseline and the 3-Month Follow-up eTable 13. Health Economic Evaluation, Baseline Outcomes and Costs (2021 USD) eTable 14. Unit Costs Used in the Costing of Resources, in 2021 USD eTable 15. Cost Means and Differences From Baseline to the 3-Month Follow-up, After Multiple Imputation, in 2021 USD eTable 16. Mean CHU9D Utility Scores per Assessment Point and Total QALYs Over the Study Period, After Multiple Imputation eTable 17. Outcomes and Costs From Baseline to the 3-Month Follow-up, After Multiple Imputation eFigure 1. Screenshots of the Internet Platform and Interventions eFigure 2. Cost-effectiveness Planes With Treatment Response as the Outcome for Three [file jamanetwopen-e2225614-s002.pdf]

Supplemental Online Content

Andrén P, Holmsved M, Ringberg H, et al. Therapist-supported internet-delivered exposure and response prevention for children and adolescents with Tourette syndrome: a randomized clinical trial. *JAMA Netw Open*. 2022;5(8):e2225614. doi:10.1001/jamanetworkopen.2022.25614

eMethods 1. Eligibility Criteria

eMethods 2. Masking Procedures

eMethods 3. Primary Outcome, Including Inter-Rater Reliability

eMethods 4. Power Analysis

eMethods 5. Further Details on the Health Economic Evaluation

eResults 1. Process Outcomes

eResults 2. Masking Integrity

eResults 3. Post hoc Analyses

eResults 4. Adverse Events

eTable 1. Additional Baseline Demographics and Clinical Characteristics for Study Participants

eTable 2. Results of Within-Group Linear Quantile Mixed Models for the Primary and Secondary Outcomes, From Baseline to the 3-Month Follow-up

eTable 3. Results of Complementary Between-Group Linear Mixed Models for the Primary and Secondary Outcomes, From Baseline to the 3-Month Follow-Up

eTable 4. Treatment Response Rates at Posttreatment (Post hoc) and the 3-Month Follow-up

eTable 5. Results of Between-Group Linear Quantile Mixed Models for Additional Secondary Outcomes

eTable 6. Results of *t* Tests and Quantile Regressions for Process Outcomes

eTable 7. Group Allocation Guesses and Motivations, by Assessors

eTable 8. Post hoc Analyses Split by Median Age, Age Group 9-11 Years

eTable 9. Post hoc Analyses Split by Median Age, Age Group 12-17 Years

© 2022 Andrén P et al. *JAMA Network Open.*

eTable 10. Post hoc Analyses Split by Gender, Boys

eTable 11. Post hoc Analyses Split by Gender, Girls

eTable 12. Reported Adverse Events Between Baseline and the 3-Month Follow-up

eTable 13. Health Economic Evaluation, Baseline Outcomes and Costs (2021 USD)

eTable 14. Unit Costs Used in the Costing of Resources, in 2021 USD

eTable 15. Cost Means and Differences From Baseline to the 3-Month Follow-up, After Multiple Imputation, in 2021 USD

eTable 16. Mean CHU9D Utility Scores per Assessment Point and Total QALYs Over the Study Period, After Multiple Imputation

eTable 17. Outcomes and Costs From Baseline to the 3-Month Follow-up, After Multiple Imputation

eFigure 1. Screenshots of the Internet Platform and Interventions

eFigure 2. Cost-effectiveness Planes With Treatment Response as the Outcome for Three Costing Perspectives

eFigure 3. Cost-effectiveness Acceptability Curves With Treatment Response as the Outcome for Three Costing Perspectives

eFigure 4. Cost-effectiveness Acceptability Curves With QALYs as the Outcome for Three Costing Perspectives

This supplemental material has been provided by the authors to give readers additional information about their work.

Therapist-Supported Internet-Delivered Exposure and Response Prevention for Children and Adolescents with Tourette Syndrome: A Randomized Clinical Trial

Per Andrén, PhD; Moa Holmsved, MSc; Helene Ringberg, MSc; Vera Wachtmeister, MSc; Kayoko Isomura, MD, PhD; Kristina Aspvall, PhD; Fabian Lenhard, PhD; Charlotte L Hall, PhD; E Bethan Davies, PhD; Tara Murphy, PhD; Chris Hollis, FRCPsych, PhD; Filipa Sampaio, PhD; Inna Feldman, PhD; Matteo Bottai, PhD; Eva Serlachius, MD, PhD; Erik Andersson, PhD; Lorena Fernández de la Cruz, PhD; David Mataix-Cols, PhD

eMethods 1. Eligibility criteria

Eligible participants were 9-to-17-year-old children with a DSM-5 diagnosis of TS or CTD[1] who had a YGTSS Total Tic Severity Score (TTSS) >15 (or >10 if only motor *or* vocal tics had been present during the last week),[2] had at least one parent available to participate in the treatment, and had access to at least one computer and one mobile phone per family. Participants were excluded if they had received ≥8 sessions of BT for tics with a qualified therapist within the past year, were receiving simultaneous psychological treatment for TS/CTD, had initiated or adjusted any psychotropic medication for TS/CTD within the past 8 weeks, had a diagnosis of organic brain disorder, intellectual disability, autism spectrum disorder, psychosis, bipolar disorder, anorexia nervosa or alcohol/substance dependence, were an immediate risk for themselves or others requiring urgent medical attention (e.g., suicidality or self-injurious tics), were not able to read and communicate in Swedish or had a close relative already enrolled in the trial.

eMethods 2. Masking procedures

The principal investigator, outcome assessors, statistician, and health economists were blind to group allocation throughout the trial. The trial coordinator was blind only during the analysis phase through the use of dummy variables for participant ID and group allocation. Participants were instructed not to reveal information about their allocated treatment to the outcome assessor. In cases where the group allocation was accidentally revealed, a new blind assessor

© 2022 Andrén P et al. *JAMA Network Open.*

watched an edited video recording of the original assessment and performed a second assessment, used in the analysis. Outcome assessors were asked to guess the participants' group allocation after each assessment.

Prior to the start of the data analyses, dummy variables for participant ID and group allocation were entered into the data files by a researcher not involved in the statistical analyses (KA). The primary outcome analysis was then independently performed by the first author (PA) and the trial statistician (MB). A meeting was then held including PA, MB, and the principal investigator (DM-C) where PA's and MB's outcomes and conclusions were compared. Both researchers had reached identical conclusions, hence consensus was reached. PA then analyzed the secondary outcomes. After the completion of all planned analyses, two separate abstracts were written and documented before revealing the true participant and group IDs (i.e., the unblinding of the study). The two abstracts included study conclusions based on the two possible options for the dummy group ID (i.e., if dummy group X was ERP and dummy group Y was the comparator, and vice versa). After revealing the true IDs, the correct abstract was selected and included in the final manuscript. Dummy variables for participant ID and group allocation were also entered into the data files used for the health economic evaluation. The health economists (FS and IF) were not informed about any of the analyses above, hence they performed their analyses independently, before unblinding.

eMethods 3. Primary outcome, including inter-rater reliability

The primary outcome was tic severity as measured by the TTSS score of the YGTSS, a clinician-rated semi-structured interview.[2] The YGTSS-TTSS comprises 10 items and yields a total score of 0-50 points, with higher scores indicating greater tic severity. All assessors were extensively trained in the use of the YGTSS (**Supplement 1**). Inter-rater reliability for the YGTSS-TTSS was calculated during the initial training of assessors using case examples (intraclass correlation coefficient; ICC [95% CI]=0.99 [0.98 to 1.00]) as well as on real cases during the study (ICC [95% CI]=0.93 [0.64 to 0.95]).

© 2022 Andrén P et al. *JAMA Network Open.*

eMethods 4. Power analysis

We estimated the power for the change in median YGTSS-TTSS between the two treatment groups from baseline to the primary endpoint with 500 bootstrap samples. To estimate the median difference, we applied a linear quantile random intercept model[3-5] with the intercept, the binary treatment variable (ERP, comparator), the numeric time variable (baseline, post-treatment, 3-month follow-up), and the treatment-by-time interaction term. For the specifications of the model, we used data from our pilot trial.[6] Based on calculations performed for the parallel Online Remote Behavioural Intervention for Tics (ORBIT) trial[7] and clinical consensus within the research team, a median difference of 3 points on the YGTSS-TTSS was judged to be the lower limit of a clinically significant change. Two hundred participants were required to detect a statistically significant change in medians of 3 points on the YGTSS-TTSS from baseline to the primary endpoint with a power of 97%. We increased the sample size to 220 patients to account for a potential 10% dropout rate. The alpha level of all tests was set at .05.

eMethods 5. Further details on the health economic evaluation

The methods of the health economic evaluation are described in the methods section of the paper and in **Supplement 1**. In this section we present additional detail on the methods.

Estimation of QALYs

Quality-adjusted life years (QALYs) were estimated by mapping KIDSCREEN-10 scores onto the multi-attribute utility instrument Child Health Utility 9 Dimensions (CHU9D)[8] using a crosswalk algorithm.[9] This was done since KIDSCREEN-10 scores cannot be directly used to derive QALYs. The CHU9D is a generic measure of health-related quality of life across nine dimensions: worried, sad, pain, tired, annoyed, schoolwork/homework, sleep, daily routine, and activities. The following algorithm was used:

© 2022 Andrén P et al. *JAMA Network Open.*

CHU9D utility = 0.222655 + (0.037867*KIDSCREEN-10_item_1) + (0.023085*KIDSCREEN-10_item_2) + (0.037192*KIDSCREEN-10_item_3) + (0.021284*KIDSCREEN-10_item_4) + (0.024877*KIDSCREEN-10_item_9) + (0.022256*KIDSCREEN-10_item_10).

The area under the curve method was used to estimate total QALY gains over the trial period.[10]

Further details on costing

Costs were estimated by multiplying frequencies by unit costs (**eTable 14**). Costs for healthcare resources were estimated using pricelists[11] and estimates from the Cost per Patient Database.[12] Medication costs were estimated using market prices.[13] Productivity losses due to absenteeism from school for children were estimated by multiplying the number of days not at school by the daily cost of a child in school. Productivity losses due to absenteeism from paid work for parents were the product of the number of days absent from work to care for the child by the average salary in Sweden (including social fees).[14] Productivity losses related to absenteeism from unpaid work corresponded to the number of days not performing unpaid work multiplied by the cost of leisure time.[15] Productivity losses related to reduced efficiency at school corresponded to the number of days at school not performing fully multiplied by a weighed score representing the reduction in productivity.[16] Total costs were calculated for each participant and aggregated over the study period. Costs were estimated in 2021 Swedish krona (SEK) and converted to 2021 U.S. dollar (USD) using Purchasing Power Parities for Gross Domestic Product.[17] No discounting was applied as all data were collected within less than one year.

Data analysis

Multiple imputation by chained equations was employed to account for missing data, assuming the economic data were likely to be missing at random.[18] Differences in costs and QALYs between groups were examined with generalized linear models[19] to allow for the consideration of other distributions to fit the data.[19] Total costs were analyzed controlling for baseline costs. Total QALYs were analyzed controlling for baseline CHU9D values.[20]

© 2022 Andrén P et al. *JAMA Network Open.*

Non-parametric bootstrapping with 5000 iterations was carried out to deal with uncertainty around the cost and outcome data. This uncertainty is presented on cost-effectiveness planes. A cost-effectiveness plane is a cloud of the 5000 bootstrapped incremental costs and effects across four quadrants, where each quadrant has a decision implication. The probability of cost-effectiveness at different willingness-to-pay thresholds is displayed on cost-effectiveness acceptability curves (CEAC).[21] The CEAC captures decision uncertainty and shows the probability of internet-delivered exposure and response prevention being cost-effective at different cost-effectiveness thresholds.[22,23]

Data was cleaned using Microsoft Excel and data analyses were performed using Stata version 15.1.

eResults 1. Process outcomes

Thirteen participants extended their treatment period from 10 to 12 weeks due to holidays (*n*=12) or sickness (*n*=1). Treatment adherence, as measured by the iiPAS, showed no differences between the groups. Self-rated need for further TS/CTD treatment was low in both groups at the 3-month follow-up, with no between-group differences. Also see **eTable 6**.

eResults 2. Masking integrity

At post-treatment, data on assessors' guesses of group allocation were available for 197 participants (221 minus 8 missing data points and 16 accidental allocation reveals), of which the assessors correctly guessed the group allocation in 113 cases (57%; $\chi 2$=4.27; p=.04). At the 3-month follow-up, there were available data for 204 participants (5 missing data points and 12 accidental reveals), with 125 correct guesses (61%; $\chi 2$=10.49; p=.001). The proportion of treatment responders at the 3-month follow-up was not different for individuals whose group membership had been guessed correctly or incorrectly (38% responders for correct guesses, 35% responders for incorrect guesses; $\chi 2$=0.10; p=.76). Similarly, the percentage of improvement on the YGTSS-TTSS between baseline and the 3-month follow-up was comparable for participants whose group membership had been guessed correctly or incorrectly (24%

improvement for correct guesses, 26% improvement for incorrect guesses; *t*=-0.51; *p*=.61). The assessors' motivations for each guess are presented in **eTable 7**.

eResults 3. Post hoc analyses

Deviating from the protocol, one participant in the comparator received 9 sessions of videoconference-delivered ERP for TS/CTD at a specialist clinic between baseline and the 3-month follow-up. In addition, 6 (3%) participants altered their TS/CTD-medication (α2-agonists or antipsychotics) between the equivalent assessment points (ERP: 1 started, 2 decreased dosage; comparator: 1 started, 1 increased dosage, 1 stopped). A linear quantile mixed model sensitivity analysis excluding these 7 participants showed a similar non-significant between-group effect on the YGTSS-TTSS as in the primary analysis (coefficient [95% CI]=-0.71 [-1.56 to 0.14]; *p*=.10).

Post-hoc analyses were performed to analyze the impact of baseline characteristics on the treatment outcome. First, the sample was split by the median age (11 years), which resulted in one group aged 9-11 (n=124) and one group aged 12-17 (n=97). A between-group linear quantile mixed model analysis identified an interaction effect between group (ERP and the comparator) and time (baseline to the 3-month follow-up) on the YGTSS-TTSS in the older group (coefficient [95% CI]=-1.21 [-2.41 to -0.02]; *p*=.05, but not in the younger group. In the older group, more participants responded to ERP (*n*=26, 51%) than the comparator (*n*=10, 23%) at the 3-month follow-up (odds ratio [95% CI]=3.43 [1.40 to 8.40]; *p*=.007). The equivalent analysis for the younger group showed no statistic difference between the groups (ERP: *n*=25, 44%; comparator: *n*=21, 32%; odds ratio [95% CI]=1.64 [0.78 to 3.42]; *p*=.19).

Second, the sample was split by gender, resulting in one group with 152 boys and one group with 68 girls (one participant was excluded due to being nonbinary). The mean age was 12.11 (SD=2.21) years for boys and 12.08 years (SD=2.35) for girls (*p*=.92). Identical linear quantile mixed model analyses to those used for the median age split found a significant interaction effect among boys (coefficient [95% CI]=-1.06 [-2.09 to -0.03]; *p*=.04), but not among girls. Among boys, significantly more participants responded to ERP (*n*=35; 51%) than

the comparator (*n*=21; 26%) at the 3-month follow-up (odds ratio [95% CI]=2.94 [1.48 to 5.84]; *p*=.002). The equivalent analysis among girls showed no between-group difference (ERP: *n*=15, 39%; comparator: *n*=10, 37%; odds ratio [95% CI]=1.11 [0.40 to 3.06]; *p*=.84). Further details on the post-hoc analyses are presented in **eTables 8-11**.

eResults 4. Adverse events

A total of 742 adverse events were recorded between baseline and the 3-month follow-up (**eTable 12**). According to the *a priori* definition (**Supplement 1**), 480 out of the 742 adverse events (65%) were expected. One treatment-unrelated serious adverse event (a case of meningitis requiring hospitalization) was recorded in the comparator group. A linear quantile mixed model found no between-group differences on the Side effects questionnaire at the 3-month follow-up (**eTable 5**).

© 2022 Andrén P et al. *JAMA Network Open.*

eTable 1. Additional baseline demographics and clinical characteristics for study participants

Characteristic, *n* (%)	ERP (*n*=111)	Comparator (*n*=110)	Total (*N*=221)
Living arrangement			
Lives with both parents	86 (77.5)	81 (73.6)	167 (75.6)
Alternating residence	17 (15.3)	19 (17.3)	36 (16.3)
Lives with one parent	7 (6.3)	10 (9.0)	17 (7.7)
Other	1 (0.9)	0 (0.0)	1 (0.5)
Referral pathway[A]			
Self-referral through study website	103 (92.8)	105 (95.5)	208 (94.1)
Clinic referral	8 (7.2)	5 (4.5)	13 (5.9)
How the family was informed about the study			
Healthcare services	54 (48.6)	61 (55.5)	115 (52.0)
Advertisement in newspaper	19 (17.1)	19 (17.3)	38 (17.2)
Website	10 (9.0)	10 (9.1)	20 (9.0)
Social media	12 (10.8)	8 (7.3)	20 (9.0)
From an acquaintance	12 (10.8)	7 (6.4)	19 (8.6)
Other	4 (3.6)	5 (4.5)	9 (4.1)
Previous contact with healthcare services mental health issues other than TS/CTD	34 (30.6)	35 (31.8)	69 (31.2)
PUTS,[24] mean (SD); range	21.45 (5.14); 9-33	20.91 (5.89); 9-36	21.18 (5.52), 9-36
SNAP-IV,[25] mean (SD); range			
ADD scale	9.16 (6.55); 0-24	8.04 (6.42); 0-27	8.60 (6.50); 0-27
Hyperactivity scale	7.03 (6.49); 0-24	6.04 (5.90); 0-26	6.53 (6.21); 0-26
ADD and hyperactivity scales combined	16.19 (11.98); 0-44	14.07 (10.92); 0-53	15.14 (11.49); 0-53
ODD scale	5.00 (4.93); 0-19	4.80 (5.41); 0-24	4.90 (5.16); 0-24
AQ-10 >5 points[B],[26]	7 (6.3)	6 (5.5)	13 (5.9)

Note: A = Healthcare services throughout Sweden were generally instructed to help interested families to self-refer to the study, rather than sending clinic referrals, due to the former saving administration time. This might explain the low number of clinic referrals despite over half of the participants were informed about the study through contact with healthcare services; B = A score >5 points is the validated cut-off for clinically significant autistic traits and may be indicative of proceeding with further autism spectrum disorder assessments.

Abbreviations: ADD = attention-deficit disorder; AQ-10 = Autism Spectrum Quotient, 10 items; comparator = therapist-supported internet-delivered education for children and adolescents with Tourette syndrome or chronic tic disorder; CTD = chronic tic disorder; ERP = therapist-supported internet-delivered exposure with response prevention for children and adolescents with Tourette syndrome or chronic tic disorder; ODD = oppositional defiant disorder; PUTS: Premonitory Urge for Tics Scale; SD = standard deviation; SNAP-IV = Swanson, Nolan, and Pelham rating scale; TS = Tourette syndrome.

© 2022 Andrén P et al. *JAMA Network Open.*

eTable 2. Results of within-group linear quantile mixed models for the primary and secondary outcomes, from baseline to the 3-month follow-up

Outcome	ERP (*n*=111)			Comparator (*n*=110)		
	Coefficient (95% CI)	*p*-value	Effect size (95% CI)[A]	Coefficient (95% CI)	*p*-value	Effect size (95% CI)[A]
YGTSS-TTSS	-3.00 (-3.63 to -2.37)	<.001*	0.60 (0.42 to 0.78)	-2.20 (-2.90 to -1.50)	<.001*	0.44 (0.28 to 0.59)
YGTSS Impairment	-5.00 (-6.85 to -3.15)	<.001*	1.00 (0.45 to 1.55)	-5.00 (-6.46 to -3.54)	<.001*	1.00 (0.46 to 1.54)
CGI-S	-0.50 (-0.83 to -0.17)	.003*	1.00 (0.26 to 1.75)	-0.02 (-0.32 to 0.27)	.88	0.05 (-0.41 to 0.51)
PTQ	-4.50 (-5.67 to -3.34)	<.001*	0.50 (0.35 to 0.65)	-4.27 (-5.80 to -2.75)	<.001*	0.47 (0.30 to 0.64)
C&A-GTS-QOL	-5.00 (-6.39 to -3.61)	<.001*	0.43 (0.28 to 0.59)	-5.16 (-6.44 to -3.88)	<.001*	0.45 (0.29 to 0.60)
CGAS	2.73 (2.12 to 3.35)	<.001*	0.50 (0.36 to 0.63)[B]	1.96 (0.99 to 2.93)	<.001*	0.36 (0.22 to 0.49)[B]
OCI-CV	-1.24 (-1.90 to -0.59)	<.001*	0.28 (0.10 to 0.45)	-0.90 (-1.48 to -0.32)	.003*	0.20 (0.07 to 0.33)
SMFQ-C	-0.42 (-0.63 to -0.22)	<.001*	0.32 (0.06 to 0.57)	-0.53 (-0.67 to -0.39)	<.001*	0.40 (0.20 to 0.60)
SMFQ-P	-0.52 (-0.70 to -0.33)	<.001*	0.39 (0.09 to 0.68)	-0.55 (-0.84 to -0.26)	<.001*	0.41 (0.18 to 0.65)
KIDSCREEN-10, child version	0.21 (-0.32 to 0.74)	.43	0.06 (-0.13 to 0.25)[B]	0.00 (-0.39 to 0.39)	>.99	0.00 (-0.14 to 0.14)[B]
KIDSCREEN-10, parent version	0.70 (0.23 to 1.17)	.003*	0.20 (0.02 to 0.38)[B]	0.33 (-0.04 to 0.71)	.08	0.10 (-0.07 to 0.26)[B]

Note: A = Bootstrapped effect sizes, interpreted as between-group differences in median relative the interquartile range, are derived from the linear quantile mixed models; B = The effect size has been inverted, so that an increase on this outcome measure is equivalent to a positive effect size, and vice versa; * = Significant at an alpha level of .05.

Abbreviations: C&A-GTS-QOL = Child and Adolescent Gilles de la Tourette Syndrome–Quality of life scale; CGAS = Children's Global Assessment Scale; CGI-S = Clinical Global Impression – Severity scale; CI = confidence interval; comparator = therapist-supported internet-delivered education for children and adolescents with Tourette syndrome or chronic tic disorder; ERP = therapist-supported internet-delivered exposure with response prevention for children and adolescents with Tourette syndrome or chronic tic disorder; Mid-treatment = 5 weeks into the treatment; OCI-CV = Obsessive-Compulsive Inventory – Child version; PTQ = Parent Tic Questionnaire; SMFQ-C = Short Mood and Feelings Questionnaire – Child version; SMFQ-P = Short Mood and Feelings Questionnaire – Parent version; SD = standard deviation; YGTSS = Yale Global Tic Severity Scale; YGTSS-TTSS: Yale Global Tic Severity Scale – Total Tic Severity Score.

© 2022 Andrén P et al. *JAMA Network Open.*

eTable 3. Results of complementary between-group linear mixed models for the primary and secondary outcomes, from baseline to the 3-month follow-up

Outcome	Intention-to-treat linear mixed model		
	Coefficient (95% CI)[A]	*p*-value	Effect size (95% CI)[A B]
YGTSS-TTSS	-0.39 (-1.08 to 0.31)	.28	0.12 (-0.11 to 0.35)
YGTSS Impairment	-0.29 (-1.39 to 0.82)	.61	0.07 (-0.20 to 0.34)
CGI-S	-0.06 (-0.16 to 0.03)	.20	0.15 (-0.08 to 0.38)
PTQ	-0.11 (-1.42 to 1.19)	.86	0.02 (-0.23 to 0.27)
C&A-GTS-QOL	0.52 (-1.23 to 2.28)	.56	-0.07 (-0.31 to 0.18)
CGAS	0.85 (0.11 to 1.59)	.02*	0.23 (0.00 to -0.45)[C]
OCI-CV	-0.34 (-1.01 to 0.33)	.32	0.11 (-0.14 to 0.37)
SMFQ-C	0.14 (-0.18 to 0.47)	.38	-0.11 (-0.39 to 0.18)
SMFQ-P	0.00 (-0.30 to 0.31)	.98	-0.00 (-0.28 to 0.27)
KIDSCREEN-10, child version	0.31 (-0.31 to 0.93)	.33	0.11 (-0.11 to 0.33)[C]
KIDSCREEN-10, parent version	0.51 (-0.06 to 1.08);	.08	0.22 (-0.04 to 0.49)[C]
Side effects questionnaire	-0.19 (-0.63 to 0.24);	.38	0.10 (-0.17 to 0.38)

Note: A = Estimates (positive or negative) compare to the comparator as the reference point; B = Bootstrapped Cohen's *d* effect sizes are derived from the linear mixed models; C = The effect size has been inverted, so that an increase on this outcome measure is equivalent to a positive effect size, and vice versa; * = Significant at an alpha level of .05.

Abbreviations: C&A-GTS-QOL = Child and Adolescent Gilles de la Tourette Syndrome–Quality of life scale; CGAS = Children's Global Assessment Scale; CGI-S = Clinical Global Impression – Severity scale; CI = confidence interval; Mid-treatment = 5 weeks into the treatment; OCI-CV = Obsessive-Compulsive Inventory – Child version; PTQ = Parent Tic Questionnaire; SMFQ-C = Short Mood and Feelings Questionnaire – Child version; SMFQ-P = Short Mood and Feelings Questionnaire – Parent version; SD = standard deviation; YGTSS = Yale Global Tic Severity Scale; YGTSS-TTSS: Yale Global Tic Severity Scale – Total Tic Severity Score.

© 2022 Andrén P et al. *JAMA Network Open.*

eTable 4. Treatment response rates at posttreatment (post hoc) and the 3-month follow-up

	ERP (*n*=111)		Comparator (*n*=110)		Logistic regression	
	n	*%*	*n*	*%*	Odds ratio (95% CI)	*p*-value
Post-treatment (*n*=213)	29	26.9	20	19.1	1.56 (0.82 to 2.98)	.18
3-month follow-up (*n*=216)	51	47.2	31	28.7	2.22 (1.27 to 3.90)	.005

Note: Treatment response is defined as a score of 1 (“Very much improved”) or 2 (“Much improved”) on the Clinical Global Impression – Improvement (CGI-I) scale.

Abbreviations: CI = confidence interval; comparator = therapist-supported internet-delivered education for children and adolescents with Tourette syndrome or chronic tic disorder; ERP = therapist-supported internet-delivered exposure with response prevention for children and adolescents with Tourette syndrome or chronic tic disorder.

© 2022 Andrén P et al. *JAMA Network Open.*

eTable 5. Results of between-group linear quantile mixed models for additional secondary outcomes

Outcome	ERP (*n*=111)		Comparator (*n*=110)		Intention-to-treat linear quantile mixed model		
	Median (IQR)[A]	Mean (SD)[A]	Median (IQR)[A]	Mean (SD)[A]	Coefficient (95% CI)[B]	*p*-value	Effect size (95% CI)[B C]
**CGAS**							
Baseline (*n*=221)	60 (55 to 65)	60.60 (6.59)	61 (56 to 65)	60.71 (6.46)	-	-	-
Post-treatment (*n*=213)	63.5 (59 to 70)	64.21 (7.90)	62 (59 to 70)	64.01 (7.59)	-	-	-
3-month follow-up[D] (*n*=216)	65 (60 to 72.5)	66.83 (8.64)	65 (59 to 70.5)	65.27 (7.44)	0.67 (-0.15 to 1.49)	.11	0.12 (-0.05 to 0.29)[E]
**OCI-CV**							
Baseline (*n*=221)	8 (4 to 13)	8.93 (6.43)	7 (3 to 12)	7.91 (5.78)	-	-	-
Post-treatment (*n*=211)	5 (2 to 9)	6.35 (5.75)	5 (1.5 to 11)	6.56 (5.82)	-	-	-
3-month follow-up[D] (*n*=207)	5 (2 to 9)	6.32 (5.87)	3.5 (1 to 10)	6.04 (6.27)	-0.28 (-1.23 to 0.68)	.57	0.06 (-0.12 to 0.24)
**SMFQ-C**							
Baseline (*n*=221)	4 (1 to 6)	4.55 (4.32)	3 (1 to 7)	4.53 (4.63)	-	-	-
Mid-treatment (*n*=212)	3 (1 to 5)	3.72 (3.87)	2 (1 to 5)	3.49 (3.61)	-	-	-
Post-treatment (*n*=211)	2 (0 to 4)	3.16 (4.44)	2 (0 to 5)	3.16 (3.52)	-	-	-
3-month follow-up[D] (*n*=207)	2 (1 to 5)	3.65 (4.51)	2 (1 to 4)	3.05 (3.29)	0.05 (-0.30 to 0.41)	.77	-0.04 (-0.30 to 0.22)
**SMFQ-P**							
Baseline (*n*=221)	3 (1 to 6)	4.44 (4.11)	3 (1 to 6)	4.21 (4.02)	-	-	-
Mid-treatment (*n*=210)	2 (1 to 4)	3.23 (3.92)	2 (1 to 5)	3.43 (3.67)	-	-	-
Post-treatment (*n*=214)	1.5 (0 to 4)	2.76 (3.80)	2 (0 to 4)	2.69 (3.02)	-	-	-
3-month follow-up[D] (*n*=210)	2 (0 to 4)	2.74 (3.66)	2 (0 to 4)	2.62 (3.07)	0.02 (-0.32 to 0.36)	.91	-0.02 (-0.26 to 0.23)
**KIDSCREEN-10, child version**							
Baseline (*n*=221)	41 (37 to 44)	40.28 (5.29)	41 (37 to 44)	40.54 (5.28)	-	-	-
Post-treatment (*n*=211)	42 (38 to 46)	41.42 (5.71)	42 (38 to 45)	40.70 (5.91)	-	-	-
3-month follow-up[D] (*n*=207)	42 (37 to 46)	40.96 (6.26)	41 (37 to 44)	40.60 (5.58)	0.10 (-0.54 to 0.75)	.75	0.03 (-0.17 to 0.23)[E]
**KIDSCREEN-10, parent version**							
Baseline (*n*=221)	41 (37 to 43)	39.96 (4.42)	40 (37 to 43)	40.28 (4.31)	-	-	-

© 2022 Andrén P et al. *JAMA Network Open.*

Post-treatment (*n*=214)	41 (37 to 43)	40.17 (4.87)	41 (38 to 44)	40.76 (4.27)	-	-	-
3-month follow-up[D] (*n*=210)	42 (38 to 45)	41.19 (4.62)	41 (38 to 44)	40.45 (4.88)	0.55 (0.07 to 1.03)	.03*	0.16 (-0.05 to 0.36)[E]
**Side effects questionnaire**							
Baseline (*n*=221)	7 (3 to 14)	8.69 (6.33)	6 (3 to 11)	7.96 (6.80)	-	-	-
Mid-treatment (*n*=210)	5 (3 to 9)	6.15 (4.80)	6 (2 to 10)	6.73 (5.44)	-	-	-
Post-treatment (*n*=214)	5 (1 to 8)	5.92 (5.90)	5 (2 to 8)	5.52 (4.25)	-	-	-
3-month follow-up[D] (*n*=210)	4 (1 to 8)	5.15 (4.87)	4 (1 to 9)	5.49 (5.22)	-0.33 (-0.85 to 0.18)	.21	0.14 (-0.08 to 0.36)

Note: A = Observed values calculated from completer data; B = Estimates (negative or positive) compare to the comparator as the reference point; C = Bootstrapped effect sizes, interpreted as between-group differences in median relative the interquartile range, are derived from the linear quantile mixed models; D = Primary endpoint; E = The effect size has been inverted, so that an increase on this outcome measure is equivalent to a positive effect size, and vice versa; * = Significant at an alpha level of .05.

Abbreviations: CGAS = Children's Global Assessment Scale; CI = confidence interval; comparator = therapist-supported internet-delivered education for children and adolescents with Tourette syndrome or chronic tic disorder; ERP = therapist-supported internet-delivered exposure with response prevention for children and adolescents with Tourette syndrome or chronic tic disorder; IQR = interquartile range; Mid-treatment = 5 weeks into the treatment; OCI-CV = Obsessive-Compulsive Inventory – Child version; SMFQ-C = Short Mood and Feelings Questionnaire – Child version; SMFQ-P = Short Mood and Feelings Questionnaire – Parent version; SD = standard deviation.

© 2022 Andrén P et al. *JAMA Network Open.*

eTable 6. Results of *t*-tests and quantile regressions for process outcomes

Outcome	ERP (*n*=111)	Comparator (*n*=110)	Statistical test	
			t-test	*p*-value
Completed chapters[A], child, mean (SD) (*n*=221)	8.85 (1.75)	8.68 (2.20)	-0.62	.54
Completed chapters[A], parent, mean (SD) (*n*=221)	8.89 (1.72)	8.82 (2.27)	-0.27	.79
Treatment completers[B], *n* (%) (*n*=221)	111 (100.0%)	104 (94.6%)	N/a[C]	
Total therapist support time, minutes per participant and week, mean (SD) (*n*=221)	19.05 (5.81)	16.55 (6.52)	-3.01	.003*
Text messages, therapist support time, minutes per participant and week, mean (SD) (*n*=221)	18.27 (5.76)	15.93 (6.36)	-2.86	.005*
Telephone, therapist support time, minutes per participant and week, mean (SD) (*n*=221)	0.78 (1.22)	0.62 (1.22)	-1.01	.32
			Quantile regression (95% CI)	*p*-value
iiPAS[D, E]				
Mid-treatment, median (IQR); mean (SD) (*n*=219)	14 (12 to 17); 14.12 (3.19)	14 (12 to 17); 13.61 (4.02)	0 (-1.44 to 1.44)	>.99
Post-treatment, median (IQR); mean (SD) (*n*=219)	13 (10 to 15); 12.59 (3.88)	12 (9.5 to 15); 11.58 (4.67)	1 (-0.44 to 2.44)	.17
Mid- + post-treatment summarized to one score, median (IQR); mean (SD) (*n*=219)	27 (23 to 31); 26.71 (6.33)	26.5 (22 to 31); 25.19 (8.00)	1 (-1.28 to 3.28)	.39
Treatment credibility[D, F], child, median (IQR); mean (SD) (*n*=216)	10 (8 to 11); 9.35 (1.70)	9 (7 to 10); 8.52 (2.21)	1 (0.38 to 1.62)	.002*
Treatment credibility[D, F], parent, median (IQR); mean (SD) (*n*=219)	10 (9 to 11); 9.68 (1.59)	9 (7 to 10); 8.51 (1.85)	1 (0.38 to 1.62)	.002*
WAI-C[D, G], median (IQR); mean (SD) (*n*=216)	40 (37 to 42); 38.28 (4.96)	37 (30 to 42); 35.48 (6.72)	3 (0.95 to 5.05)	.004*
WAI-P[D, G], median (IQR); mean (SD) (*n*=219)	41 (38 to 42); 39.34 (3.74)	39.5 (33 to 42); 36.17 (7.48)	2 (-0.05 to 4.05)	.06
Treatment satisfaction[D, H], child, median (IQR); mean (SD) (*n*=206)	26 (23 to 29); 25.40 (5.14)	23 (19 to 26); 22.04 (5.49)	3 (1.13 to 4.87)	.002*
Treatment satisfaction[D, H], parent, median (IQR); mean (SD) (*n*=210)	27 (23 to 29); 25.98 (4.34)	23 (20 to 28); 23.38 (5.19)	4 (2.33 to 5.66)	<.001*
Need for further TS/CTD treatment[I], child, median (IQR); mean (SD) (*n*=207)	1 (0 to 2); 1.30 (1.11)	1 (0 to 2); 1.28 (1.17)	0 (-0.42 to 0.42)	>.99

© 2022 Andrén P et al. *JAMA Network Open.*

Need for further TS/CTD treatment[I], parent, median (IQR); mean (SD) (*n*=210)	2 (1 to 2); 1.63 (1.12)	2 (1 to 3); 1.90 (1.34)	0 (-0.62 to 0.62)	>.99

Note: A = Range 0-10 chapters; B = Completion of child chapters 1 to 4; C = Not applicable because 100% in one of the two groups; D = Higher scores indicates higher treatment adherence, treatment credibility, working alliance and treatment satisfaction, respectively; E = 5 items, range 0-20 points; F = 3 items, range 0 to 12 points; G = 6 items, range 0 to 42 points; H = Composite score of items 1 to 8, range 0 to 32 points; I = 1 item, range 0 to 4 points, from 0 = "I do not need any more treatment for my tics" to 4 = "I need very much more treatment for my tics"; * = Significant at an alpha level of .05.

Abbreviations: comparator = therapist-supported internet-delivered education for children and adolescents with Tourette syndrome or chronic tic disorder; CTD = chronic motor or vocal tic disorder; ERP = therapist-supported internet-delivered exposure with response prevention for children and adolescents with Tourette syndrome or chronic tic disorder; IQR = interquartile range; Mid-treatment = 5 weeks into the treatment; SD = standard deviation; TS = Tourette syndrome; WAI-C = Working Alliance Inventory – Child version; WAI-P = Working Alliance Inventory – Parent version.

© 2022 Andrén P et al. *JAMA Network Open.*

eTable 7. Group allocation guesses and motivations, by assessors

	Post-treatment		3-month follow-up	
	**Frequency**	**Percentage**	**Frequency**	**Percentage**
**Correctly guessed group allocation**	113	57.4%	125	61.3%
**Correctly guessed ERP group**	53	26.9%	61	29.9%
Motivation/reason for guess				
The participant improved	31	58.5%	41	67.2%
Pure guess	19	35.8%	19	31.1%
Other specified reason	3	5.7%	1	1.6%
**Correctly guessed the comparator**	60	30.5%	64	31.4%
Motivation/reason for guess				
Pure guess	35	58.3%	45	70.3%
The participant did not improve	22	36.7%	18	28.1%
Other specified reason	3	5.0%	1	1.6%
**Incorrectly guessed ERP group**	45	22.8%	43	21.1%
Motivation/reason for guess				
Pure guess	23	51.1%	22	51.2%
The participant improved	19	42.2%	21	48.8%
Other specified reason	3	6.7%	-	-
**Incorrectly guessed the comparator**	39	19.8%	36	17.6%
Motivation/reason for guess				
Pure guess	22	56.4%	25	69.4%
The participant did not improve	15	38.5%	11	30.6%
Other specified reason	2	5.1%	-	-

Abbreviations: comparator = therapist-supported internet-delivered education for children and adolescents with Tourette syndrome or chronic tic disorder; ERP = therapist-supported internet-delivered exposure with response prevention for children and adolescents with Tourette syndrome or chronic tic disorder.

© 2022 Andrén P et al. *JAMA Network Open.*

eTable 8. Post hoc analyses split by median age, age group 9-11 years

Outcome	ERP (*n*=65)		Comparator (*n*=59)		Linear quantile mixed model		
	Median (IQR)[A]	Mean (SD)[A]	Median (IQR)[A]	Mean (SD)[A]	Coefficient (95% CI)[B]	*p*-value	Effect size (95% CI)[B C]
**YGTSS-TTSS**							
Baseline (*n*=124)	24 (20 to 26)	23.46 (4.79)	24 (21 to 27)	23.49 (5.50)	-	-	-
Post-treatment (*n*=123)	19 (16 to 23)	19.38 (5.80)	20 (14 to 23)	18.63 (7.21)	-	-	-
3-month follow-up[D] (*n*=122)	18 (14 to 22)	17.30 (6.28)	19 (11 to 23)	16.88 (7.36)	-0.02 (-1.51 to 1.48)	.98	0.00 (-0.29 to 0.30)

Outcome	ERP (*n*=65)		Comparator (*n*=59)		Logistic regression	
	n	%	*n*	%	Odds ratio (95% CI)	*p*-value
**Treatment response[E]**						
3-month follow-up[D] (*n*=122)	25	43.9	21	32.3	1.64 (0.78 to 3.42)	.19

Note: A = Observed values calculated from completer data; B = Estimates (negative or positive) compare to the comparator as the reference point; C = Bootstrapped effect sizes, interpreted as between-group differences in median relative the interquartile range, are derived from the linear quantile mixed models; D = Primary endpoint; E = Treatment response is defined as a score of 1 (“Very much improved”) or 2 (“Much improved”) on the Clinical Global Impression – Improvement (CGI-I) scale; * = Significant at an alpha level of .05.

Abbreviations: CI = confidence interval; comparator = therapist-supported internet-delivered education for children and adolescents with Tourette syndrome or chronic tic disorder; ERP = therapist-supported internet-delivered exposure with response prevention for children and adolescents with Tourette syndrome or chronic tic disorder; IQR = interquartile range; SD = standard deviation; YGTSS-TTSS: Yale Global Tic Severity Scale – Total Tic Severity Score.

© 2022 Andrén P et al. *JAMA Network Open.*

eTable 9. Post hoc analyses split by median age, age group 12-17 years

Outcome	ERP (*n*=52)		Comparator (*n*=45)		Linear quantile mixed model		
	Median (IQR)[A]	Mean (SD)[A]	Median (IQR)[A]	Mean (SD)[A]	Coefficient (95% CI)[B]	*p*-value	Effect size (95% CI)[B C]
**YGTSS-TTSS**							
Baseline (*n*=97)	21 (15 to 25)	20.88 (6.16)	23 (17 to 27)	22.31 (6.48)	-	-	-
Post-treatment (*n*=90)	17.5 (13 to 23)	17.54 (6.01)	19.5 (15 to 26)	20.30 (7.15)	-	-	-
3-month follow-up[D] (*n*=94)	14 (10 to 20)	14.90 (7.23)	19 (15 to 24)	19.00 (6.59)	-1.21 (-2.41 to -0.02)	.05*	0.22 (-0.05 to 0.49)

Outcome	ERP (*n*=52)		Comparator (*n*=45)		Logistic regression	
	n	%	*n*	%	Odds ratio (95% CI)	*p*-value
**Treatment response[E]**						
3-month follow-up[D] (*n*=94)	26	51.0	10	23.3	3.43 (1.40 to 8.40)	.007*

Note: A = Observed values calculated from completer data; B = Estimates (negative or positive) compare to the comparator as the reference point; C = Bootstrapped effect sizes, interpreted as between-group differences in median relative the interquartile range, are derived from the linear quantile mixed models; D = Primary endpoint; E = Treatment response is defined as a score of 1 (“Very much improved”) or 2 (“Much improved”) on the Clinical Global Impression – Improvement (CGI-I) scale; * = Significant at an alpha level of .05.

Abbreviations: CI = confidence interval; comparator = therapist-supported internet-delivered education for children and adolescents with Tourette syndrome or chronic tic disorder; ERP = therapist-supported internet-delivered exposure with response prevention for children and adolescents with Tourette syndrome or chronic tic disorder; IQR = interquartile range; SD = standard deviation; YGTSS-TTSS: Yale Global Tic Severity Scale – Total Tic Severity Score.

© 2022 Andrén P et al. *JAMA Network Open.*

eTable 10. Post hoc analyses split by gender, boys

Outcome	ERP (*n*=71)[A]		Comparator (*n*=81)		Linear quantile mixed model		
	Median (IQR)[B]	Mean (SD)[B]	Median (IQR)[B]	Mean (SD)[B]	Coefficient (95% CI)[C]	*p*-value	Effect size (95% CI)[C D]
**YGTSS-TTSS**							
Baseline (*n*=152)	22 (18 to 26)	22.10 (5.69)	24 (20 to 27)	23.01 (5.91)	-	-	-
Post-treatment (*n*=148)	18 (13 to 22)	18.10 (6.09)	20.5 (15 to 24)	19.60 (6.65)	-	-	-
3-month follow-up[E] (*n*=150)	16 (10 to 21)	15.28 (7.01)	19 (13 to 23)	17.96 (6.87)	-1.06 (-2.09 to -0.03)	.04*	0.21 (-0.01 to 0.44)

Outcome	ERP (*n*=71)[A]		Comparator (*n*=81)		Logistic regression	
	n	%	*n*	%	Odds ratio (95% CI)	*p*-value
**Treatment response[F]**						
3-month follow-up[E] (*n*=150)	35	50.7	21	25.9	2.94 (1.48 to 5.84)	.002*

Note: A = One participant in the ERP group was excluded from these analyses due to being nonbinary; B = Observed values calculated from completer data; C = Estimates (negative or positive) compare to the comparator as the reference point; D = Bootstrapped effect sizes, interpreted as between-group differences in median relative the interquartile range, are derived from the linear quantile mixed models; E = Primary endpoint; F = Treatment response is defined as a score of 1 (“Very much improved”) or 2 (“Much improved”) on the Clinical Global Impression – Improvement (CGI-I) scale; * = Significant at an alpha level of .05.

Abbreviations: CI = confidence interval; comparator = therapist-supported internet-delivered education for children and adolescents with Tourette syndrome or chronic tic disorder; ERP = therapist-supported internet-delivered exposure with response prevention for children and adolescents with Tourette syndrome or chronic tic disorder; IQR = interquartile range; SD = standard deviation; YGTSS-TTSS: Yale Global Tic Severity Scale – Total Tic Severity Score.

© 2022 Andrén P et al. *JAMA Network Open.*

eTable 11. Post hoc analyses split by gender, girls

Outcome	ERP (*n*=39)[A]		Comparator (*n*=29)		Linear quantile mixed model		
	Median (IQR)[B]	Mean (SD)[B]	Median (IQR)[B]	Mean (SD)[B]	Coefficient (95% CI)[C]	*p*-value	Effect size (95% CI)[C D]
**YGTSS-TTSS**							
Baseline (*n*=68)	23 (20 to 25)	22.26 (5.29)	24 (19 to 27)	23.00 (6.07)	-	-	-
Post-treatment (*n*=64)	19 (16 to 23)	19.03 (5.44)	19 (13 to 24)	18.30 (8.66)	-	-	-
3-month follow-up[E] (*n*=65)	18 (13 to 21)	17.53 (6.20)	16 (10 to 23)	17.00 (7.88)	0.30 (-1.28 to 1.88)	.71	-0.06 (-0.30 to 0.42)

Outcome	ERP (*n*=39)[A]		Comparator (*n*=29)		Logistic regression	
	n	%	*n*	%	Odds ratio (95% CI)	*p*-value
**Treatment response[F]**						
3-month follow-up[E] (*n*=65)	15	39.5	10	37.0	1.11 (0.40 to 3.06)	.84

Note: A = One participant in the ERP group was excluded from these analyses due to being nonbinary; B = Observed values calculated from completer data; C = Estimates (negative or positive) compare to the comparator as the reference point; D = Bootstrapped effect sizes, interpreted as between-group differences in median relative the interquartile range, are derived from the linear quantile mixed models; E = Primary endpoint; F = Treatment response is defined as a score of 1 (“Very much improved”) or 2 (“Much improved”) on the Clinical Global Impression – Improvement (CGI-I) scale; * = Significant at an alpha level of .05.

Abbreviations: CI = confidence interval; comparator = therapist-supported internet-delivered education for children and adolescents with Tourette syndrome or chronic tic disorder; ERP = therapist-supported internet-delivered exposure with response prevention for children and adolescents with Tourette syndrome or chronic tic disorder; IQR = interquartile range; SD = standard deviation; YGTSS-TTSS: Yale Global Tic Severity Scale – Total Tic Severity Score.

© 2022 Andrén P et al. *JAMA Network Open.*

eTable 12. Reported adverse events between baseline and the 3-month follow-up

Adverse event[A]	ERP (*n*=111)			Comparator (*n*=110)		
	Number	Expected[B]	Treatment related[C] Yes/No/Uncertain	Number	Expected[B]	Treatment related[C] Yes/No/Uncertain
Increased tics	88	Yes	4/10/74	73	Yes	2/4/67
Irritability/anger/outbursts/disruptive behavior	68	Yes	8/1/59	63	Yes	2/3/58
Sick, cold, influenza, COVID-19, etc.	32	No	0/32/0	20[D]	No	0/20/0
Depressed mood	31	Yes	11/6/14	42	Yes	7/7/28
Suicidal ideation	23	No	0/2/21	13	No	0/0/13
Sleep problems	21	Yes	0/1/20	30	Yes	0/0/30
Anxiety/worry/stress	18	Yes	8/3/7	13	Yes	4/1/8
Excitement[E]	17	No	0/0/17	14	No	0/0/14
Decreased appetite	12	No	0/1/11	13	No	0/0/13
Tiredness/fatigue/drowsiness	10	Yes	2/1/7	9	Yes	0/3/6
Increased isolation	9	No	1/0/8	6	No	0/0/6
Bullying	9	No	0/9/0	6	No	1/5/0
Stomachache	8	No	0/2/6	12	No	0/6/6
Conflicts with family and/or peers	8	No	5/0/3	4	No	1/2/1
Headache	7	Yes	0/2/5	7	Yes	0/0/7
Nightmares	6	No	0/0/6	3	No	0/0/3
Obsessions and/or compulsions	5	No	0/1/4	3	No	0/1/2
Pain[F]	5	No	3/0/2	2	No	0/0/2
Physical harm/injury	3	No	0/0/3	8	No	1/4/3
Staring and/or daydreaming	3	No	0/0/3	0	No	0/0/0
Restless	2	No	1/0/1	1	No	0/0/1
Self-harm	2	No	0/0/2	1	No	0/1/0

© 2022 Andrén P et al. *JAMA Network Open.*

Motor problems	1	No	0/0/1	3	No	0/1/2
Hopelessness	1	No	0/0/1	2	No	1/0/1
Other	2	No	1/0/1	3	No	2/0/1
**Total**	**391**	**243 (62.1%)**	**44/71/276**	**351**	**237 (67.5%)**	**21/58/272**

Note: A = Adverse event data have been combined from the following sources: therapist/researcher communication (manual log kept from baseline to the 3-month follow-up), SMFQ-C total score (<13 points, if greater than baseline score), SMFQ additional suicide item (<1 point, if greater than baseline score), Side effects questionnaire (any item ≥2 points ["about half the time"] if greater than baseline score). The SMFQ-C, the additional suicide item, and the Side effects questionnaire were all collected at baseline, 5 weeks into the treatment, at post-treatment, and at the 3-month follow-up. More information about the adverse event reporting is found in the full study protocol (Supplement 1); B = Each adverse event is listed as expected or unexpected according to an *a priori* definition in the full study protocol (Supplement 1), which in turn is based on data from our pilot study; C = Each adverse event is reported as treatment related, treatment unrelated, or uncertain treatment relation. The categorization of treatment relation is based on information reported by the families and by clinical judgement among the researchers. In many cases however, there were insufficient data to safely categorize, hence the "uncertain treatment relation" category was used; D = One serious adverse event (judged to be treatment unrelated) was reported in the study, a case of meningitis in the comparator group. This specific adverse event is reported in the category "Sick/cold/influenza/COVID-19 etc."**;** E = Excitement is not necessarily an unwanted event but is included in this table because it is an item on the Side effects questionnaire; F = The item pain excludes headaches, stomachaches, physical harm/injury, and self-harm, which all are reported separately.

Abbreviations: comparator = therapist-supported internet-delivered education for children and adolescents with Tourette syndrome or chronic tic disorder; COVID-19 = Coronavirus disease 2019; ERP = therapist-supported internet-delivered exposure with response prevention for children and adolescents with Tourette syndrome or chronic tic disorder.

© 2022 Andrén P et al. *JAMA Network Open.*

eTable 13. Health economic evaluation, baseline outcomes and costs (2021 USD)

Outcomes and costs	ERP (*n*=111)	Comparator (*n*=109)[A]
	Mean (SD)	Mean (SD)
**Outcomes**		
KIDSCREEN-10, child version	39.96 (4.42)	40.21 (4.27)
CHU9D (Utility)[B]	0.869 (0.105)	0.872 (0.098)
**Costs**[C]		
Total healthcare costs	505.74 (522.88)	537.81 (668.83)
Total societal costs	1167.81 (1767.46)	879.86 (1435.86)

Note: A = One participant in the comparator was excluded from the health economic evaluation due to having been hospitalized (sometime between baseline and the primary endpoint) following a treatment-unrelated serious adverse event. Cost estimates for this hospitalization was uncertain and it was judged that the inclusion of these costs could have skewed the between-group comparison; B = CHU9D utilities were estimated from KIDSCREEN-10 (child-reported version) scores using a mapping algorithm; C = Costs refer to a 3-month timeframe prior to the participants were recruited to the study, hence excluding the costs for the study interventions. Costs are uprated to 2021 and converted from SEK to USD, where relevant, using Purchasing Power Parities.[17]

Abbreviations: CHU9D = Child Health Utility 9D; comparator = therapist-supported internet-delivered education for children and adolescents with Tourette syndrome or chronic tic disorder; ERP = therapist-supported internet-delivered exposure with response prevention for children and adolescents with Tourette syndrome or chronic tic disorder; SD = standard deviation.

© 2022 Andrén P et al. *JAMA Network Open.*

eTable 14. Unit costs used in the costing of resources, in 2021 USD

Resource item	Unit cost (USD)[A]	Source
**Healthcare resources (per visit)**		
Counsellor	229.25	Region Stockholm and Sweden’s Municipalities and Regions[B]
Dietician	313.38	Sweden’s Municipalities and Regions
General practitioner	203.27	Region Stockholm
Nurse	205.76	Region Stockholm and Sweden’s Municipalities and Regions[B]
Speech and language therapist	336.87	Sweden’s Municipalities and Regions
Specialist practitioner[C]	477.17	Sweden’s Municipalities and Regions
Physiotherapist	195.82	Sweden’s Municipalities and Regions
Psychologist	272.39	Region Stockholm and Sweden’s Municipalities and Regions[B]
Other treatments (e.g., acupuncture, osteopathy)	67.76	Market prices
**Medication**		
Over the counter supplements	Individual product prices	Market price from the Swedish pharmacy chain Apotea[13]
Prescription drugs	Individual product prices	The Dental and Pharmaceutical Benefits Agency of Sweden
**Support and assistance**		
Host family (per day)	80.07	Sweden’s Municipalities and Regions
Special assistant (per hour)	35.57	Swedish Insurance Agency
Special teacher (per hour)	45.06	Own estimate
Study buddy (per hour)	51.71	Own estimate
**Productivity losses**		
Average wage/hour in Sweden[D]	36.53	Statistics Sweden
Cost of leisure time/hour[D]	17.84	Post-tax wage/hour in Sweden[15]
Cost per child/day at school	79.72	Own estimate based on Swedish National Agency for Education
**Intervention cost**		
Internet-delivered BT therapist average wage (per hour)	36.96	Project documentation

© 2022 Andrén P et al. *JAMA Network Open.*

Note: A = Costs are uprated to 2021 and converted from SEK to USD, where relevant, using Purchasing Power Parities;[17] B = Corresponds to an average of the cost for primary care and specialist care; C = Based on an average of 11 medical specialties; D = Includes social fees of 43.3%.[27]

© 2022 Andrén P et al. *JAMA Network Open.*

eTable 15. Cost means and differences from baseline to the 3-month follow-up,[A] after multiple imputation, in 2021 USD

	ERP (*n*=111)		Comparator (*n*=109)[B]		Mean difference			
**Type of cost, from the TiC-P**	Mean	SE	Mean	SE	Unadjusted mean diff.	Adjusted mean diff.[C]	95% LCI	95% UCI
Healthcare visits	703.03	162.95	649.10	106.45	53.93	92.91	-54.72	605.22
Medication/supplements	89.81	23.47	49.94	9.32	39.86	46.32	4.16	168.16
Social support and assistance	244.33	145.42	225.64	95.56	18.69	32.10	-64.49	1351.21
Parental paid productivity loss[D]	678.40	186.37	641.47	125.33	36.93	85.37	-87.33	784.71
Parental unpaid productivity loss[E]	583.52	209.63	582.03	149.96	1.49	21.71	-102.60	866.52
School support	563.53	334.13	147.54	62.86	415.99	253.37	-11.10	3536.00
Child school absenteeism	462.08	72.79	555.62	62.66	-93.54	-68.07	-108.65	25.60
Child school presenteeism	137.88	64.41	132.47	44.78	5.41	-32.20	-34.86	173.46
**Costs summed per perspective**								
Intervention costs (healthcare organization perspective)[F]	117.38	8.78	102.23	3.65	15.14	-	5.08	25.20
Total healthcare costs (healthcare sector perspective)[G]	910.33	170.72	801.35	106.70	108.98	91.30	-64.48	452.31
Total societal costs (societal perspective)[H]	3580.52	669.47	3088.16	409.55	492.35	26.56	-404.46	976.75

Note: A = The TiC-P data in eTable 15 were collected at post-treatment and at the 3-month follow-up. Since the TiC-P asks about the last 3 months, the total recall period was close to being from baseline to the 3-month follow-up (10 weeks from baseline to post-treatment, and 3 months from post-treatment to the 3-month follow-up). B = One participant in the comparator was excluded from the health economic evaluation due to having been hospitalized (sometime between baseline and the primary endpoint) following a treatment-unrelated serious adverse event. Cost estimates for this hospitalization was uncertain and it was judged that the inclusion of these costs could have skewed the between-group comparison; C = Adjusted mean differences calculated using generalized linear models (GLM) adjusted for baseline cost, except for the intervention costs; D = Work absenteeism; E = Housework; F = Costs of the ERP or comparator interventions; G = Costs of the ERP or comparator interventions, healthcare visits, and medication/supplements; H = Costs of the ERP or comparator interventions, healthcare visits, medication/supplements, and societal costs (e.g., productivity losses, child school absenteeism).

© 2022 Andrén P et al. *JAMA Network Open.*

Abbreviations: comparator = therapist-supported internet-delivered education for children and adolescents with Tourette syndrome or chronic tic disorder; ERP = therapist-supported internet-delivered exposure with response prevention for children and adolescents with Tourette syndrome or chronic tic disorder; LCI = lower 95% confidence interval; SE = standard error; TiC-P = Trimbos/iMTA questionnaire for costs associated with psychiatric illness; UCI = upper 95% confidence interval.

© 2022 Andrén P et al. *JAMA Network Open.*

eTable 16. Mean CHU9D utility scores per assessment point and total QALYs over the study period, after multiple imputation

**Outcome**	**ERP (*n*=111)**			**Comparator (*n*=109)**[A]		
	Mean	SE	95% CI	Mean	SE	95% CI
**CHU9D** (Utility)[B]						
Baseline	0.869	0.010	0.850-0.889	0.873	0.009	0.854-0.891
Post-treatment	0.894	0.010	0.874-0.914	0.879	0.010	0.859-0.898
3-month follow-up	0.877	0.011	0.855-0.898	0.872	0.010	0.852-0.892
**Total QALYs**	0.392	0.004	0.384-0.399	0.388	0.004	0.381-0.395

Note: A = One participant in the comparator was excluded from the health economic evaluation due to having been hospitalized (sometime between baseline and the primary endpoint) following a treatment-unrelated serious adverse event. Cost estimates for this hospitalization was uncertain and it was judged that the inclusion of these costs could have skewed the between-group comparison; B = CHU9D utilities were estimated from KIDSCREEN-10 (child-reported version) scores using a mapping algorithm.

Abbreviations: CHU9D = Child Health Utility 9D; CI = confidence interval; comparator = therapist-supported internet-delivered education for children and adolescents with Tourette syndrome or chronic tic disorder; ERP = therapist-supported internet-delivered exposure with response prevention for children and adolescents with Tourette syndrome or chronic tic disorder; QALY = quality-adjusted life year; SE = standard error.

© 2022 Andrén P et al. *JAMA Network Open.*

eTable 17. Outcomes and costs from baseline to the 3-month follow-up,[A] after multiple imputation

	ERP (*n*=111)		Comparator (*n*=109)[B]		Mean difference					
**Outcomes**	Mean	SE	Mean	SE	Unadjusted mean diff.	Adjusted mean diff.[C]	95% LCI	95% UCI	ICER Treatment response[D]	ICER QALY[D]
Total QALYs	0.392	0.011	0.388	0.005	0.004	0.003	-0.004	0.010	-	-
Treatment response[E]	0.472	-	0.280	-	0.192	-	0.068	0.316	-	-
**Costs summed per perspective**[F]										
Intervention costs (healthcare organization perspective)[G]	117.38	8.78	102.23	3.65	15.14	-	5.08	25.20	79	5496
Total healthcare costs (healthcare sector perspective)[H]	910.33	170.72	801.35	106.70	108.98	91.30	-64.48	452.31	476	33138
Total societal costs (societal perspective)[I]	3580.52	669.47	3088.16	409.55	492.35	26.56	-404.46	976.75	138	9641

Note: A = The TiC-P data in eTable 15 were collected at post-treatment and at the 3-month follow-up. Since the TiC-P asks about the last 3 months, the total recall period was close to being from baseline to the 3-month follow-up (10 weeks from baseline to post-treatment, and 3 months from post-treatment to the 3-month follow-up). B = One participant in the comparator was excluded from the health economic evaluation due to having been hospitalized (sometime between baseline and the primary endpoint) following a treatment-unrelated serious adverse event. Cost estimates for this hospitalization was uncertain and it was judged that the inclusion of these costs could have skewed the between-group comparison; C = Adjusted mean differences calculated using generalized linear models (GLM) adjusted for baseline CHU9D scores in the analysis of QALYs and baseline cost in the analyses of costs (except for the intervention costs); D = Bootstrapped adjusted values; E = Treatment response is defined as a score of 1 (“Very much improved”) or 2 (“Much improved”) on the Clinical Global Impression – Improvement (CGI-I) scale; F = Costs are uprated to 2021 and converted from SEK to USD, where relevant, using Purchasing Power Parities;[17] G = Costs of the ERP or comparator interventions; H = Costs of the ERP or comparator interventions, healthcare visits, and medication/supplements; I = Costs of the ERP or comparator interventions, healthcare visits, medication/supplements, and societal costs (e.g., productivity losses, child school absenteeism).

© 2022 Andrén P et al. *JAMA Network Open.*

Abbreviations: CHU9D = Child Health Utility 9D; comparator = therapist-supported internet-delivered education for children and adolescents with Tourette syndrome or chronic tic disorder; ERP = therapist-supported internet-delivered exposure with response prevention for children and adolescents with Tourette syndrome or chronic tic disorder; ICER = incremental cost-effectiveness ratio; LCI = lower 95% confidence interval; QALY = quality-adjusted life year; SE = standard error; UCI = upper 95% confidence interval.

© 2022 Andrén P et al. *JAMA Network Open.*

eFigure 1. Screenshots of the internet platform and interventions

1. Overview and start page, with list of chapters. This screenshot is taken from the ERP intervention, but both interventions share the same appearance.

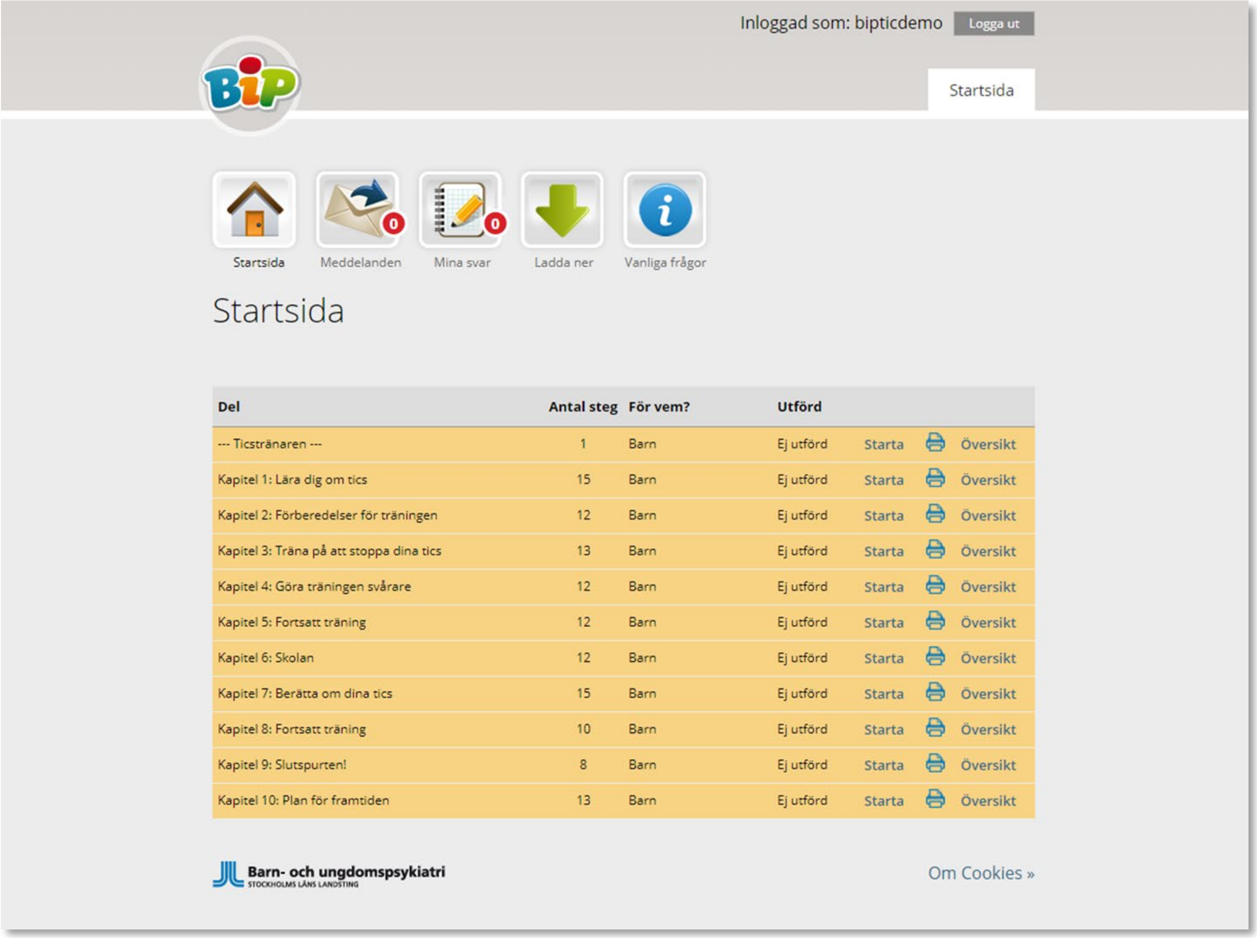

© 2022 Andrén P et al. *JAMA Network Open.*

2. Encrypted messaging function, where the participant can send messages to the therapist. This function is used in both interventions.

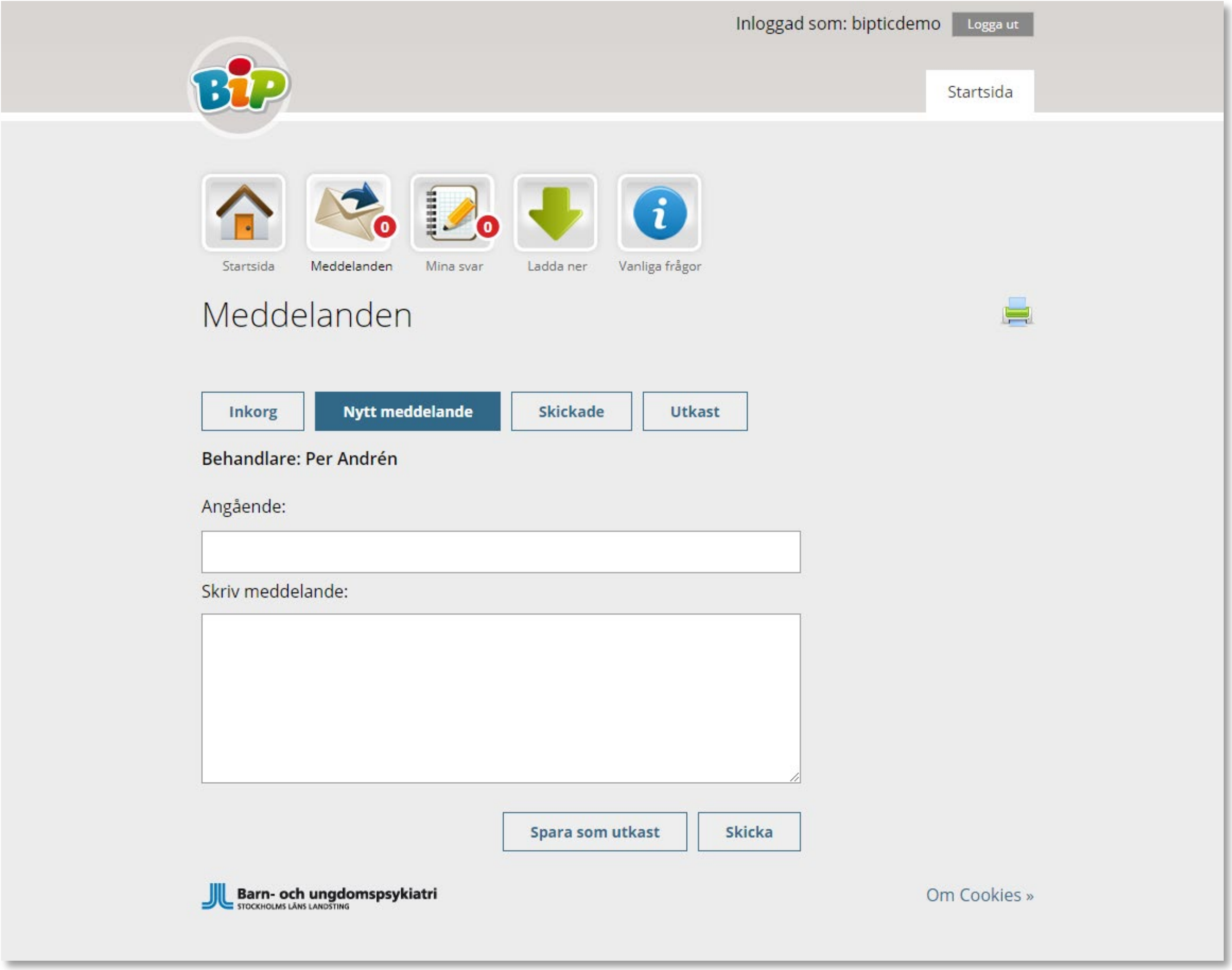

3. Educational films, used for providing psychoeducation and the treatment rationale. This format is used in both interventions.

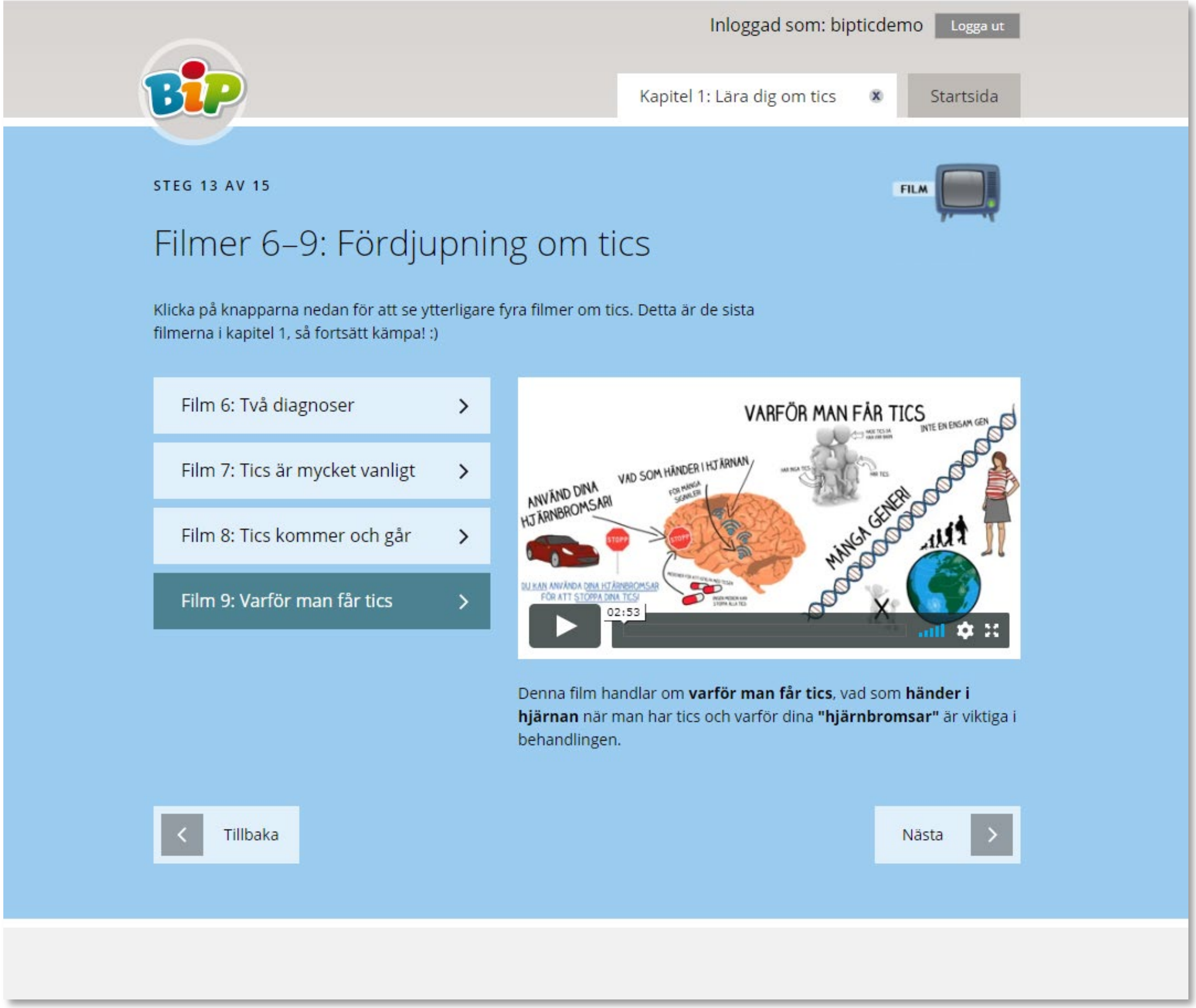

4. Worksheets and exercises. This format is used in both interventions.

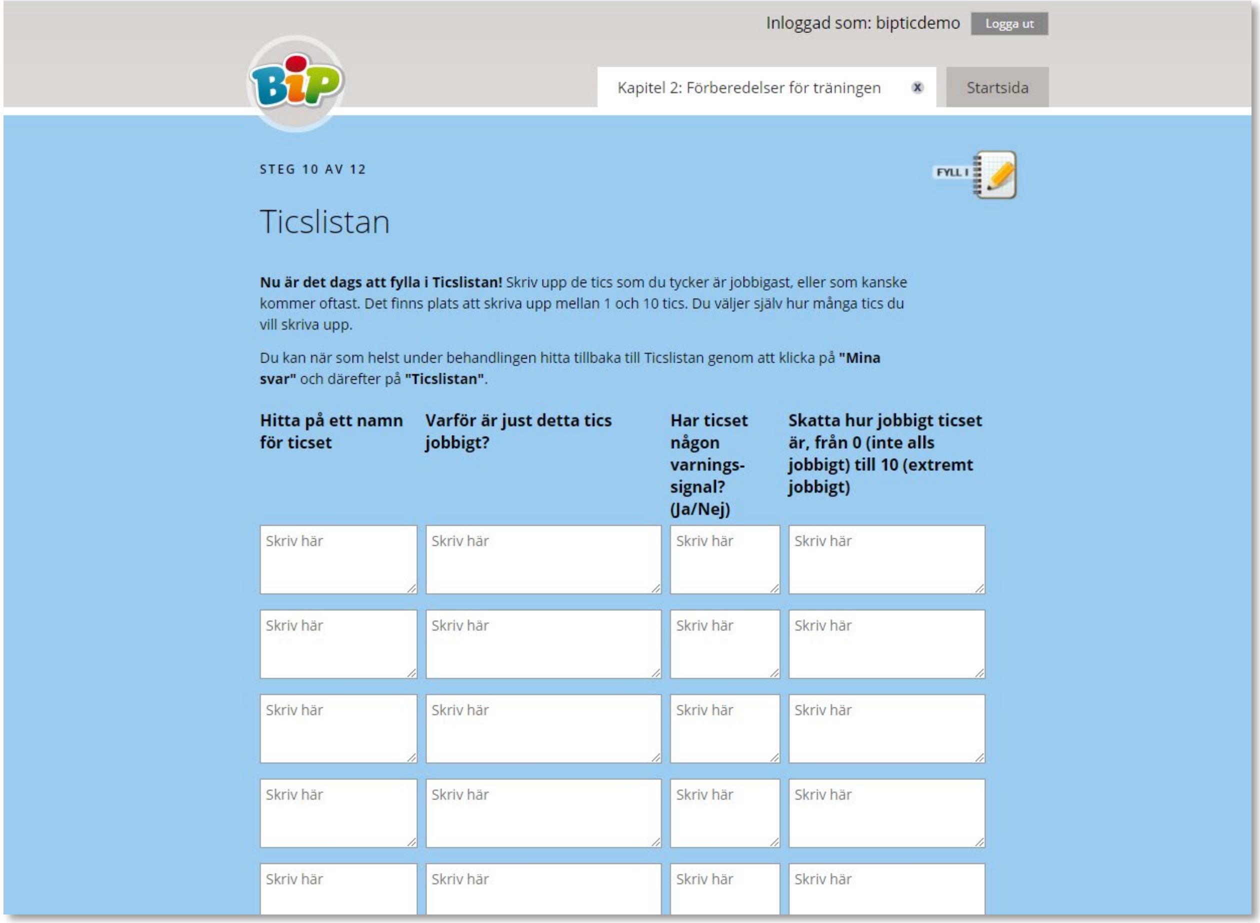

© 2022 Andrén P et al. *JAMA Network Open.*

5. Stopwatch with a high score function, for use during tic suppression. The stopwatch is used only in the ERP intervention.

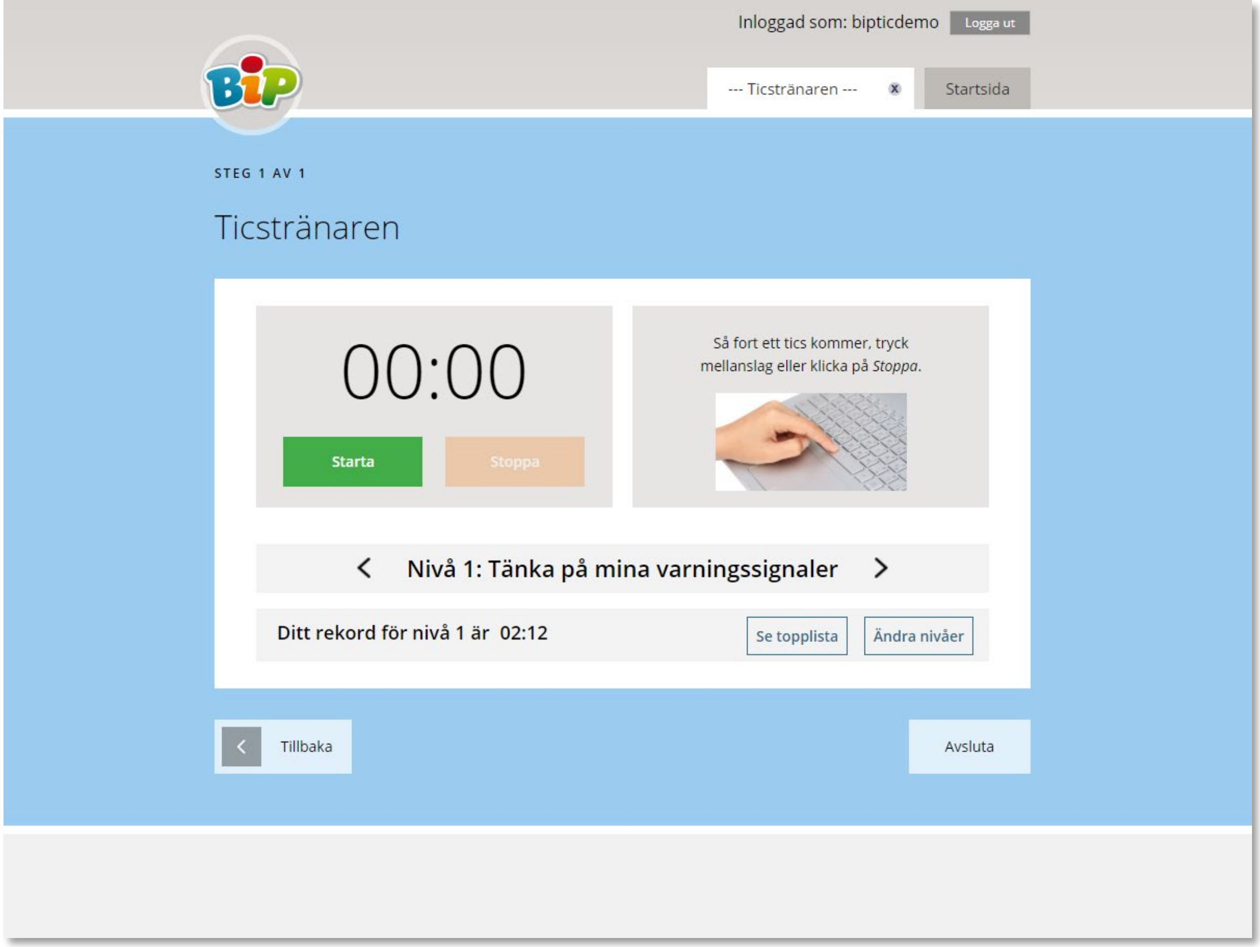

© 2022 Andrén P et al. *JAMA Network Open.*

eFigure 2. Cost-effectiveness planes with treatment response as the outcome for three costing perspectives

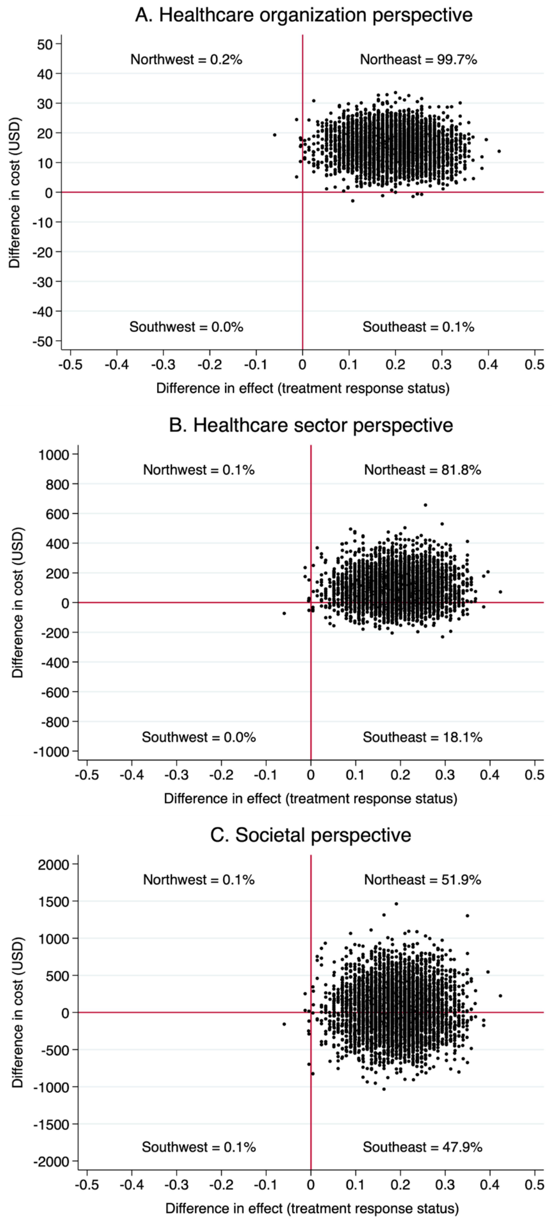

Note: All three cost-effectiveness planes compare ERP to the comparator using treatment response status as the outcome. Treatment response is defined as a score of 1 (“Very much improved”) or 2 (“Much improved”) on the Clinical Global Impression – Improvement (CGI-I) scale. The three planes only differ by the costing perspective. The healthcare organization perspective (A) includes costs of the ERP or comparator interventions (i.e., the therapist-support time). The healthcare sector perspective (B) includes costs of the ERP or comparator interventions, healthcare visits, and medication/supplements. The societal perspective (C) includes costs of the ERP or comparator interventions, healthcare visits, medication/supplements, and other sector costs (e.g., productivity losses, child school absenteeism). The probability of the ERP group showing higher treatment response rates at higher costs (northeast quadrant) is 99.7% in A, 81.8% in B, and 51.9% in C. The equivalent

probability of the ERP group showing higher treatment response rates at lower costs (southeast quadrant) is 0.1% in A, 18.1% in B, and 47.9% in C. Overall, there is not much probability for the ERP group to show lower treatment response rates, at any costing perspective (northwest and southwest quadrants).

Abbreviations: comparator = therapist-supported internet-delivered education for children and adolescents with Tourette syndrome or chronic tic disorder; ERP = therapist-supported internet-delivered exposure with response prevention for children and adolescents with Tourette syndrome or chronic tic disorder.

© 2022 Andrén P et al. *JAMA Network Open.*

eFigure 3. Cost-effectiveness acceptability curves with treatment response as the outcome for three costing perspectives

A. Healthcare organization perspective

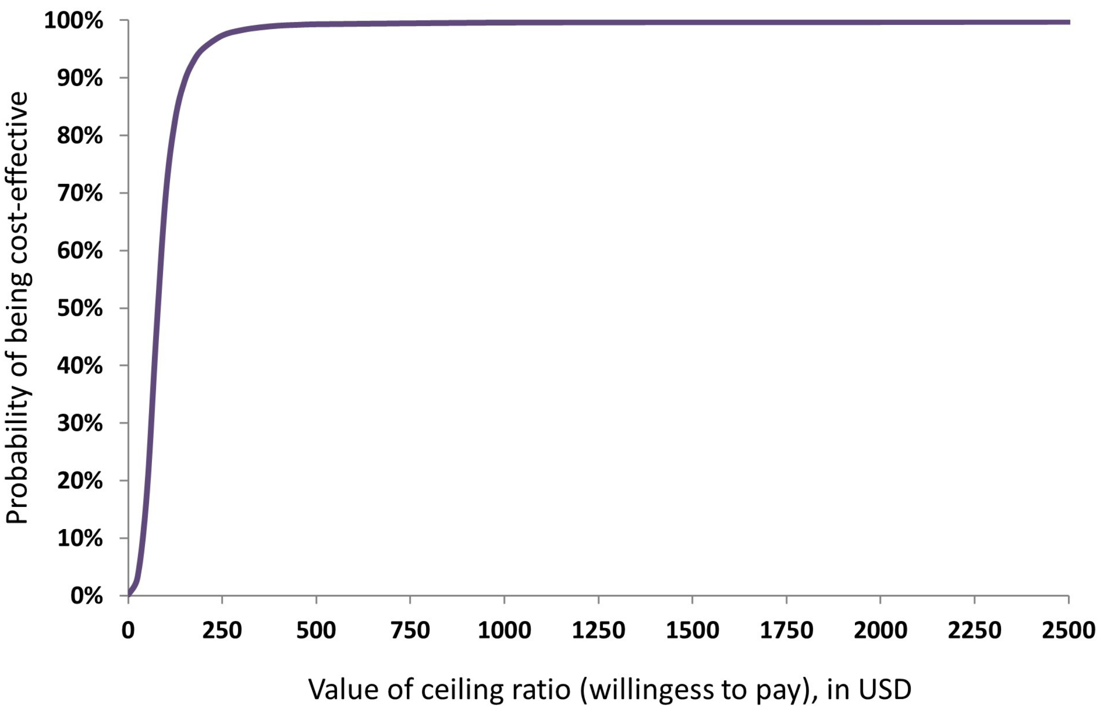

B. Healthcare sector perspective

© 2022 Andrén P et al. *JAMA Network Open.*

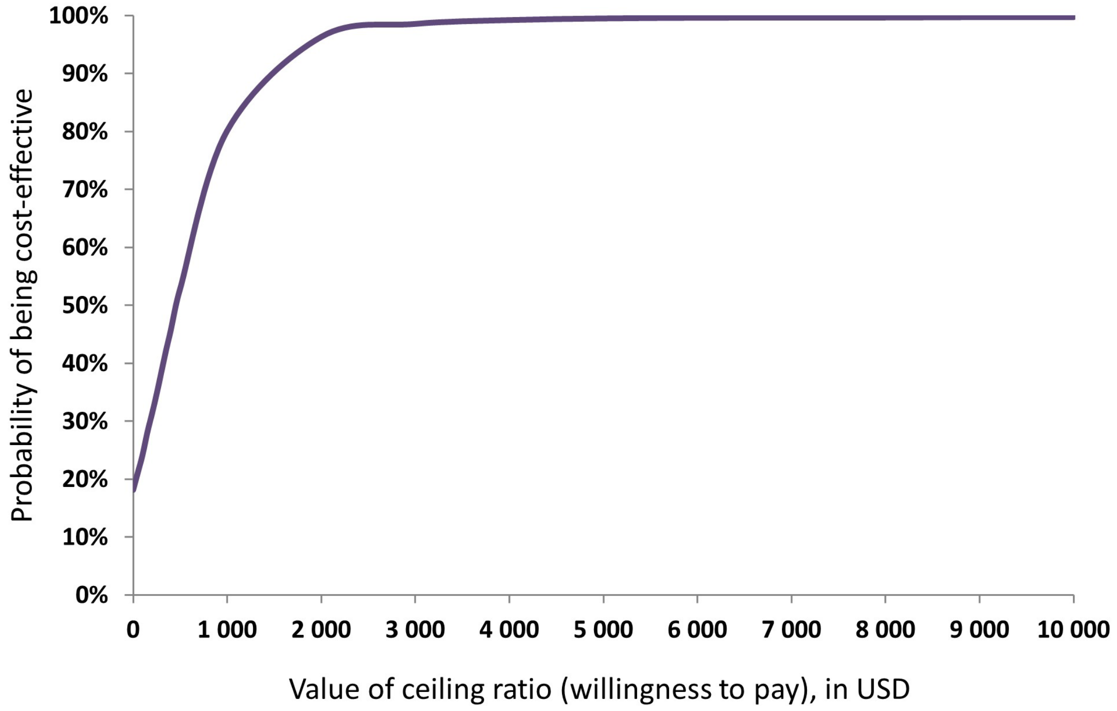

C. Societal perspective

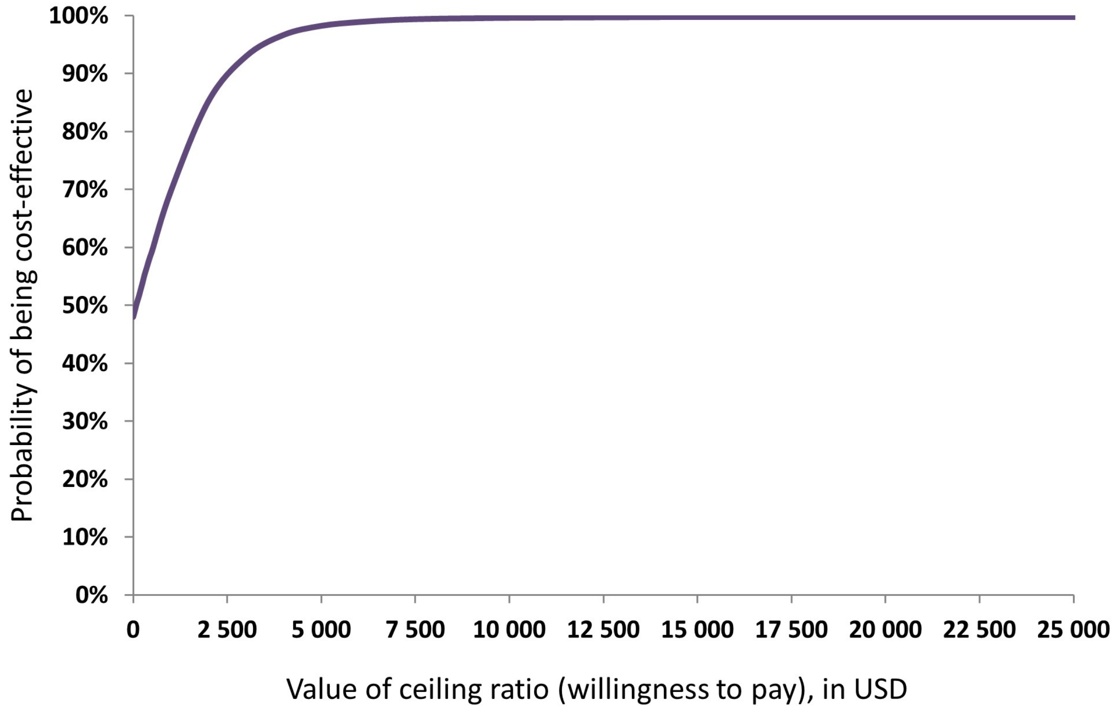

Note: The cost-effectiveness acceptability curves show the probability for the ERP intervention to be cost-effective at different willingness-to-pay levels (in USD), with treatment response as the outcome, for three costing perspectives. Treatment response is defined as a score of 1 ("Very much improved") or 2 ("Much improved") on the Clinical Global Impression – Improvement

© 2022 Andrén P et al. *JAMA Network Open.*

(CGI-I) scale. The healthcare organization perspective (A) includes costs of the ERP or comparator interventions (i.e., the therapist-support time). The healthcare sector perspective (B) includes costs of the ERP or comparator interventions, healthcare visits, and medication/supplements. The societal perspective (C) includes costs of the ERP or comparator interventions, healthcare visits, medication/supplements, and other sector costs (e.g., productivity losses, child school absenteeism).

Abbreviations: comparator = therapist-supported internet-delivered education for children and adolescents with Tourette syndrome or chronic tic disorder; ERP = therapist-supported internet-delivered exposure with response prevention for children and adolescents with Tourette syndrome or chronic tic disorder.

eFigure 4. Cost-effectiveness acceptability curves with QALYs as the outcome for three costing perspectives

A. Healthcare organization perspective

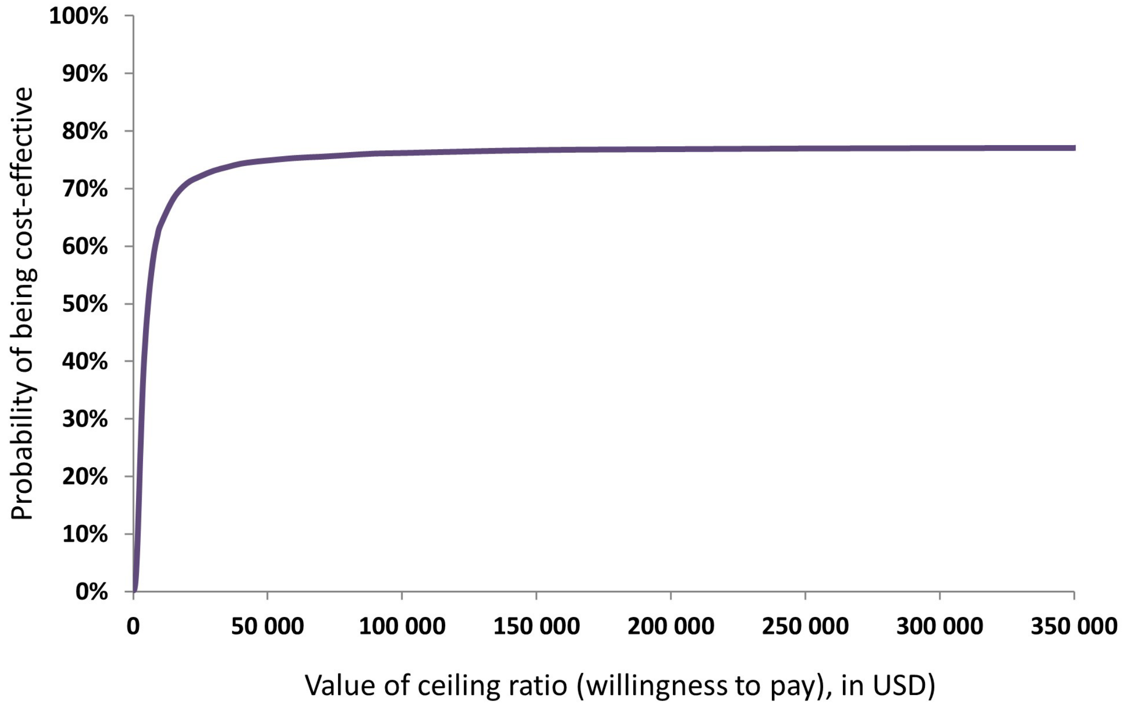

B. Healthcare sector perspective

© 2022 Andrén P et al. *JAMA Network Open.*

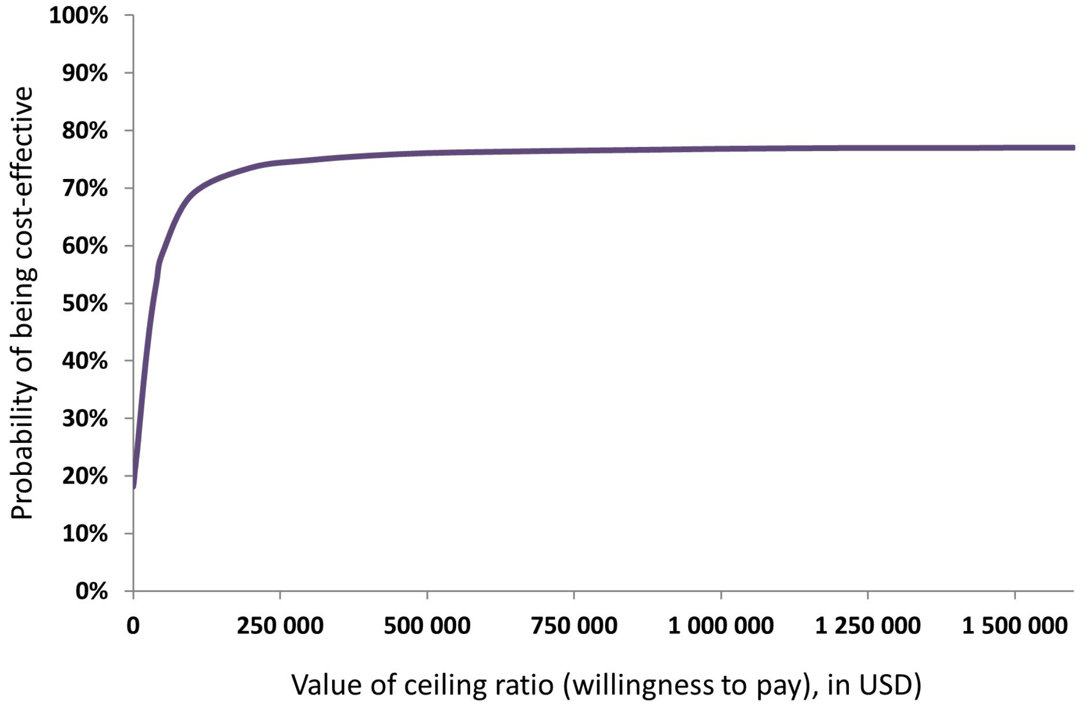

C. Societal perspective

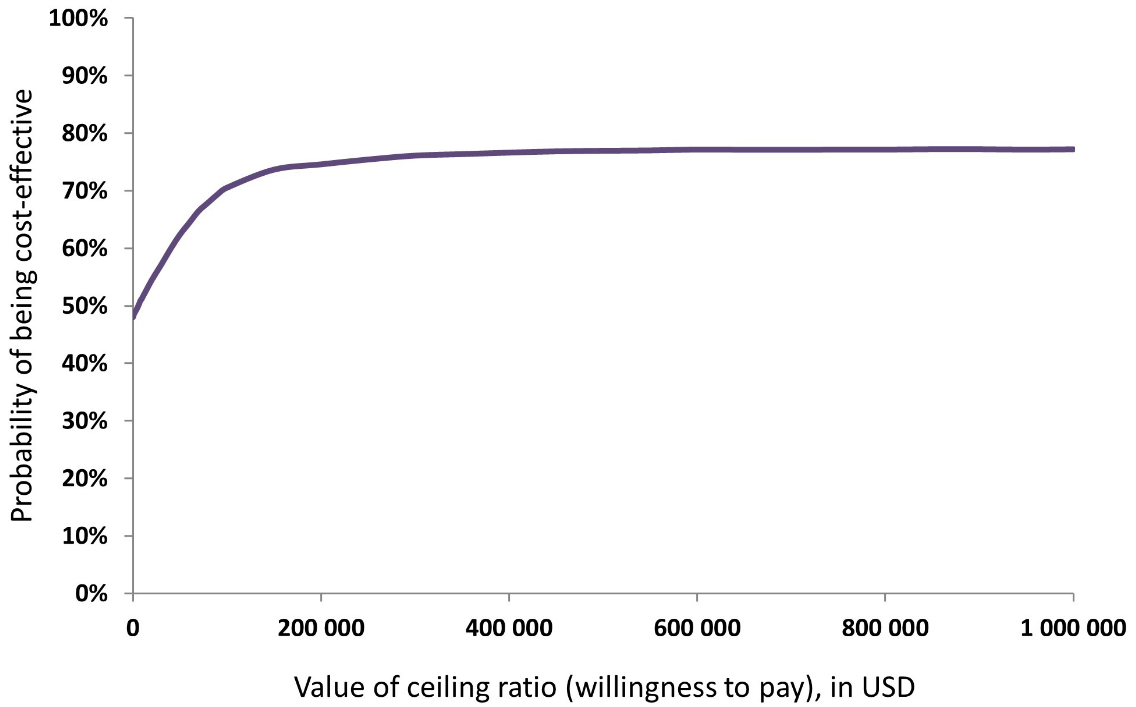

Note: The cost-effectiveness acceptability curves show the probability for the ERP intervention to be cost-effective at different willingness-to-pay levels (in USD), with QALYs as the outcome, for three costing perspectives. The healthcare organization

© 2022 Andrén P et al. *JAMA Network Open.*

perspective (A) includes costs of the ERP or comparator interventions (i.e., the therapist-support time). The healthcare sector perspective (B) includes costs of the ERP or comparator interventions, healthcare visits, and medication/supplements. The societal perspective (C) includes costs of the ERP or comparator interventions, healthcare visits, medication/supplements, and other sector costs (e.g., productivity losses, child school absenteeism).

Abbreviations: comparator = therapist-supported internet-delivered education for children and adolescents with Tourette syndrome or chronic tic disorder; ERP = therapist-supported internet-delivered exposure with response prevention for children and adolescents with Tourette syndrome or chronic tic disorder; QALY = quality-adjusted life year.

© 2022 Andrén P et al. *JAMA Network Open.*

References

1. American Psychiatric Association. *Diagnostic and Statistical Manual of Mental Disorders: DSM-5.* 5th ed. Washington, D.C.: American Psychiatric Publishing; 2013.
2. Leckman JF, Riddle MA, Hardin MT, et al. The Yale Global Tic Severity Scale: initial testing of a clinician-rated scale of tic severity. *J Am Acad Child Adolesc Psychiatry.* 1989;28(4):566-573.
3. Geraci M, Bottai M. Quantile regression for longitudinal data using the asymmetric Laplace distribution. *Biostatistics.* 2007;8(1):140-154.
4. Geraci M, Bottai M. Linear quantile mixed models. *Stat Comput.* 2014;24(3):461-479.
5. Liu Y, Bottai M. Mixed-Effects Models for Conditional Quantiles with Longitudinal Data. *International Journal of Biostatistics.* 2009;5(1).
6. Andrén P, Aspvall K, Fernández de la Cruz LW, P., et al. Therapist-guided and parent-guided internet-delivered behaviour therapy for paediatric Tourette's disorder: a pilot randomised controlled trial with long-term follow-up. *BMJ Open.* 2019;9.
7. Hall CL, Davies EB, Andrén P, et al. Investigating a therapist-guided, parent-assisted remote digital behavioural intervention for tics in children and adolescents-'Online Remote Behavioural Intervention for Tics' (ORBIT) trial: protocol of an internal pilot study and single-blind randomised controlled trial. *BMJ Open.* 2019;9(1):e027583.
8. Stevens K. Assessing the performance of a new generic measure of health-related quality of life for children and refining it for use in health state valuation. *Appl Health Econ Health Policy.* 2011;9(3):157-169.
9. Chen G, Stevens K, Rowen D, Ratcliffe J. From KIDSCREEN-10 to CHU9D: creating a unique mapping algorithm for application in economic evaluation. *Health Qual Life Outcomes.* 2014;12:134.
10. Matthews JN, Altman DG, Campbell MJ, Royston P. Analysis of serial measurements in medical research. *BMJ.* 1990;300(6719):230-235.
11. Region Stockholm. https://www.regionstockholm.se/sthlm-gotland Accessed 21 March, 2022.
12. Swedish Association of Local Authorities and Regions. https://skr.se/skr/halsasjukvard/ekonomiavgifter/kostnadperpatientkpp.1076.html. Accessed 21 March, 2022.
13. Apotea. https://www.apotea.se/. Accessed 23 November, 2021.
14. Statistics Sweden (Statistiska centralbyrån). https://www.scb.se/hitta-statistik/sverige-i-siffror/lonesok/. Accessed 23 November, 2021.
15. Neumann PJ, Ganiats TG, Russell LB, Sanders GD, Siegel JE. *Cost-Effectiveness in Health and Medicine.* 2 ed. New York: Oxford University Press; 2016.
16. Kigozi J, Jowett S, Lewis M, Barton P, Coast J. The Estimation and Inclusion of Presenteeism Costs in Applied Economic Evaluation: A Systematic Review. *Value Health.* 2017;20(3):496-506.
17. Organisation for Economic Co-operation and Development (Purchasing Power Parities). . http://eppi.ioe.ac.uk/costconversion/. Accessed 23 November, 2021.
18. Schafer JL, Graham JW. Missing data: our view of the state of the art. *Psychol Methods.* 2002;7(2):147-177.
19. Barber J, Thompson S. Multiple regression of cost data: use of generalised linear models. *J Health Serv Res Policy.* 2004;9(4):197-204.
20. Manca A, Hawkins N, Sculpher MJ. Estimating mean QALYs in trial-based cost-effectiveness analysis: the importance of controlling for baseline utility. *Health Econ.* 2005;14(5):487-496.
21. Drummond M, Sculpher M, Torrance G, O'Brien B, Stoddart G. *Methods for the economic evaluation of health care programmes.* 2005.

© 2022 Andrén P et al. *JAMA Network Open.*

22. Fenwick E, Claxton K, Sculpher M. Representing uncertainty: the role of cost-effectiveness acceptability curves. *Health Econ.* 2001;10(8):779-787.
23. Drummond MF, Sculpher MJ, Claxton K, Stoddart GL, Torrance GW. *Methods for the Economic Evaluation of Health Care Programmes.* Oxford: Oxford: Oxford University Press; 2015.
24. Woods DW, Piacentini J, Himle MB, Chang S. Premonitory Urge for Tics Scale (PUTS): initial psychometric results and examination of the premonitory urge phenomenon in youths with Tic disorders. *Journal of Developmental & Behavioral Pediatrics.* 2005;26(6):397-403.
25. Bussing R, Fernandez M, Harwood M, et al. Parent and teacher SNAP-IV ratings of attention deficit hyperactivity disorder symptoms: psychometric properties and normative ratings from a school district sample. *Assessment.* 2008;15(3):317-328.
26. Allison C, Auyeung B, Baron-Cohen S. Toward brief "Red Flags" for autism screening: The Short Autism Spectrum Quotient and the Short Quantitative Checklist for Autism in toddlers in 1,000 cases and 3,000 controls [corrected]. *J Am Acad Child Adolesc Psychiatry.* 2012;51(2):202-212 e207.
27. Ekonomifakta. https://www.ekonomifakta.se/fakta/skatter/skatt-pa-arbete/sociala-avgifter-over-tid. Accessed 23 November, 2021.
