# Supplementary material for: Therapist-Supported Internet-Delivered Exposure and Response Prevention for Children and Adolescents With Tourette Syndrome: A Randomized Clinical Trial
Source: JAMA Netw Open. 2022 Aug 15;5(8):e2225614. doi: 10.1001/jamanetworkopen.2022.25614 (PMC9379743; doi:10.1001/jamanetworkopen.2022.25614)
Supplement: Supplement 3. — Data Sharing Statement [file jamanetwopen-e2225614-s003.pdf]

Andrén P, Holmsved M, Ringberg H, et al. Therapist-supported internet-delivered exposure and response prevention for children and adolescents with Tourette syndrome. *JAMA Netw Open*. 2022;5(8):e2225614. doi:10.1001/jamanetworkopen.2022.25614

Data Sharing Statement

Data

Data available: No

Additional Information

Explanation for why data not available: The data are pseudonymised according to national (Swedish) and European Union legislation and cannot be anonymised and published in an open repository. Participants in the trial consent for their data to be shared with other international researchers for research purposes. The data can be made available upon reasonable request on a case-by-case basis according to the current legislation and ethical permits.
